# Applying the Estimands Framework to Non‐Inferiority Trials: Guidance on Choice of Hypothetical Estimands for Non‐Adherence and Comparison of Estimation Methods

**DOI:** 10.1002/sim.10348

**Published:** 2025-02-07

**Authors:** Katy E. Morgan, Ian R. White, Clémence Leyrat, Simon Stanworth, Brennan C. Kahan

**Affiliations:** ^1^ Department of Medical Statistics London School of Hygiene & Tropical Medicine London UK; ^2^ MRC Clinical Trials Unit UCL London UK; ^3^ Radcliffe Department of Medicine University of Oxford Oxford UK

**Keywords:** estimand, instrumental variables, intercurrent event, inverse probability weighting, non‐inferiority trial

## Abstract

A common concern in non‐inferiority (NI) trials is that non‐adherence due, for example, to poor study conduct can make treatment arms artificially similar. Because intention‐to‐treat analyses can be anti‐conservative in this situation, per‐protocol analyses are sometimes recommended. However, such advice does not consider the estimands framework, nor the risk of bias from per‐protocol analyses. We therefore sought to update the above guidance using the estimands framework, and compare estimators to improve on the performance of per‐protocol analyses. We argue the main threat to validity of NI trials is the occurrence of “trial‐specific” intercurrent events (IEs), that is, IEs which occur in a trial setting, but would not occur in practice. To guard against erroneous conclusions of non‐inferiority, we suggest an estimand using a hypothetical strategy for trial‐specific IEs should be employed, with handling of other non‐trial‐specific IEs chosen based on clinical considerations. We provide an overview of estimators that could be used to estimate a hypothetical estimand, including inverse probability weighting (IPW), and two instrumental variable approaches (one using an informative Bayesian prior on the effect of standard treatment, and one using a treatment‐by‐covariate interaction as an instrument). We compare them, using simulation in the setting of all‐or‐nothing compliance in two active treatment arms, and conclude both IPW and the instrumental variable method using a Bayesian prior are potentially useful approaches, with the choice between them depending on which assumptions are most plausible for a given trial.

## 1 Introduction

Non‐inferiority (NI) trials aim to show a new treatment is not worse than a standard treatment by more than a pre‐defined amount (the non‐inferiority margin) [1, 2, 3]. NI trials are often used in settings where a new treatment may not improve outcomes compared to a standard treatment but is expected to have other benefits such as reduced cost or an improved safety profile.

A longstanding concern in NI trials is that non‐adherence to assigned treatment due to poor trial conduct can make treatment arms appear more similar than they would be in practice [2, 4, 5, 6, 7]. This artificial similarity can increase the risk of declaring non‐inferiority when using intention‐to‐treat (ITT) analyses (i.e., an analysis in which all participants are followed up and their observed outcomes are analyzed according to their assigned treatment arm), even when the new intervention is worse than the standard treatment. For these reasons, major guidelines have historically recommended that ITT analyses be supplemented with per‐protocol analyses which exclude non‐adherent participants, as this analysis is assumed to be less affected by deviations due to poor study conduct [6, 7]. However, per‐protocol analyses do not correspond to a well‐defined treatment effect and can be biased due to post‐baseline exclusions. Importantly, the bias can either increase or decrease the risk of falsely declaring non‐inferiority, depending on the pattern of protocol deviations [8, 9, 10]. Attention in recent years has therefore focussed on identifying more suitable estimators which rely on less stringent assumptions [5, 6, 11, 12].

However, with the recent publication of the ICH‐E9(R1) addendum, there is growing recognition that investigators should start with the estimand (the treatment effect they wish to estimate), and then choose an estimator aligned to this estimand [13, 14, 15, 16, 17, 18, 19, 20]. Thus, there is urgent need to update the standard guidance on analyses of NI trials based on the estimands framework, and to identify appropriate estimators for the chosen estimands. A key component of defining an estimand is specifying how intercurrent events (post randomization events which affect the interpretation or existence of outcome data, such as non‐adherence to assigned treatment or treatment discontinuation) are handled. An ITT analysis in which all patients' outcomes are observed and included in the analysis, regardless of whether they experienced the intercurrent event, typically targets an estimand where all intercurrent events are handled using a *treatment policy* strategy, where the event is taken to be part of the treatment condition and thus considered irrelevant [19]. However, this strategy may not always reflect the most important clinical question. Further, it is less clear what estimand strategy a per‐protocol analysis corresponds to, whether additional estimands would always be necessary in NI trials, or how best to estimate the appropriate estimands.

Given the uncertainty around both the appropriate application of estimands to NI trials and the most appropriate estimators, we sought to (i) discuss how the estimands framework can be applied to non‐inferiority trials; and (ii) compare different methods of estimating hypothetical estimands for NI trials with non‐compliance in two active treatment arms. The paper is structured as follows: in Section 2 we provide a motivating example, and in Section 3 we give recommendations for applying the estimands framework to non‐inferiority trials. Although the focus of this paper is non‐inferiority trials, some of the guidance we give in Section 3 could also be applied to superiority trials if trial‐specific intercurrent events are anticipated. In Section 4 we provide a formal definition of the recommended estimand using the potential outcomes framework, and in Section 5 we provide an overview of different estimators that could be used for the recommended estimand. In Sections 6 and 7 we provide the methods and results of a simulation study evaluating the different estimators, and in Section 8 we provide a re‐analysis of our motivating example. In Section 9 we consider how our framework could be applied to a prospective trial. We conclude in Section 10 with a discussion. A summary of key points is given in Box 1. The primary focus of this paper is on choice of estimands and choice of estimators for non‐inferiority trials, thus we do not discuss issues such as how the non‐inferiority margin should be chosen (except to note that such choices should involve consideration of the estimand). In this paper we use “non‐adherence,” “non‐compliance,” and “occurrence of an intercurrent event” as synonymous. In our setting, with a one‐off treatment, by “non‐adherence” or “non‐compliance” we mean that patients were assigned to a certain treatment but did not receive it.

BOX 1 **Summary of key points.**

- In non‐inferiority trials, non‐inferiority conclusions are in reference to a specific estimand. For instance, treatment A may be non‐inferior to treatment B if rescue medication is allowed (i.e., for the comparison “treatment A + rescue medication as needed” versus “treatment B + rescue medication as needed”), but not if rescue medication were withheld (i.e., for the comparison of “treatment A without rescue” versus “treatment B without rescue”). Clarification of the estimand(s) on which non‐inferiority will be assessed is essential for proper interpretation of trial results.
- In general, intercurrent events such as treatment discontinuation or use of rescue medication do not pose a threat to the validity of non‐inferiority trials. For instance, if interest lies in the comparison of “treatment A + rescue medication as needed” versus “treatment B + rescue medication as needed,” the intercurrent event of rescue medication does not increase the risk of a spurious non‐inferiority finding.
- An exception to this is the occurrence of trial‐specific intercurrent events (see Box 2). These intercurrent events can make the treatment groups within the study artificially similar, which *can* increase the risk of a spurious non‐inferiority finding.
- Trial‐specific intercurrent events can manifest in different ways (see Box 2), and may not always be identifiable from the trial data. Thus, they require careful consideration when choosing an estimand, choosing an estimator, and interpreting trial results (see Table 2 for recommendations).
- Trial‐specific intercurrent events can also pose a threat to the validity of superiority trials, although the implications are different (i.e., they may increase the chances of a false non‐inferiority finding in non‐inferiority trials, while they may decrease the chances of finding superiority in superiority trials). However, in both cases they increase the risk of an incorrect interpretation, and as such they warrant consideration for any trial design.

## 2 Motivating Example: The TOPPS Trial

This work was motivated by the TOPPS (Trial of Prophylactic Platelets) trial, which two authors were involved in (BCK, SS) [21]. TOPPS was a randomized non‐inferiority trial comparing two different platelet transfusion policies in patients with hematologic cancers. It assessed whether a non‐prophylactic transfusion policy (new treatment; patients only received a platelet transfusion if they showed signs of any bleeding) was non‐inferior to a prophylactic transfusion strategy (standard treatment; patients received a platelet transfusion if their platelet count dropped below 10 × 10 [9] per liter) to prevent major bleeding. The primary outcome was occurrence of at least one WHO grade 2–4 bleed within 30 days of randomization. The non‐inferiority margin was a difference of 15 percentage points, meaning that a non‐prophylactic strategy could be considered acceptable for use in practice if it did not increase the number of patients experiencing a bleed by more than 15 percentage points. The main perceived benefits of a non‐prophylactic approach were lower risk of transfusion related adverse events and substantial cost savings.

The main intercurrent event was deviation from the allocated transfusion policy by administering a platelet transfusion against protocol. The primary analysis followed an ITT strategy where patients were included regardless of whether they deviated from their assigned transfusion policy or not, and was supplemented with a secondary per‐protocol analysis which excluded participants with any deviations from their allocated transfusion policy. Full details are available in the original publication [21].

However, contrary to conventional wisdom, the per‐protocol analysis was in fact less conservative than the ITT analysis. While the ITT analysis did not support non‐inferiority (adjusted difference in percentage points of 8.4, 90% CI 1.7 to 15.2), the per‐protocol analysis did show non‐inferiority of the non‐prophylactic approach (adjusted difference 4.5, 90% CI −3.0 to 12.0) (Table S1). This discrepancy between analyses likely occurred due to confounding in the per‐protocol analysis, where a much higher proportion of patients who experienced a bleeding event were excluded from the non‐prophylactic group compared to the prophylactic group (Table S2). This result highlights the need to identify and adopt estimators which rely on less stringent assumptions than per‐protocol analyses.

## 3 Recommendations for Applying the Estimands Framework to Non‐Inferiority Trials

In Table 1, we describe the difference in philosophy around the implications of poor adherence to assigned treatment (or other intercurrent events) in non‐inferiority trials from a statistical versus estimands perspective. We argue that non‐adherence or protocol deviations themselves are not inherently a threat to the validity of non‐inferiority trials. Many such intercurrent events occur in routine clinical practice, and are thus simply something that needs to be defined as part of the estimand based on clinical considerations, as in any other trial design.

**TABLE 1:** Issue of poor adherence in non‐inferiority trials from a statistical versus estimands perspective.

Statistical perspective	Estimands perspective
Treatment deviations due to poor study conduct or other reasons can make treatment arms more similar than they would otherwise be. In superiority trials affected by this, ITT is “conservative” (less likely to show a statistically significant effect). However, in affected NI trials, ITT is “anti‐conservative” (more likely to demonstrate non‐inferiority, even when the new treatment is in fact worse). Per‐protocol analyses, which exclude participants with such deviations, have been argued to be more conservative than ITT analyses in these settings (though this is not always true), and thus are often performed alongside ITT analyses to help protect against false conclusions of NI based on poor study conduct.	“Trial‐specific” intercurrent events (those which occur in a trial setting but not in routine practice, for instance due to poor trial conduct, clinician decisions due to uncertainties about the evidence base, etc.) can make treatment arms more similar in terms of actual treatment received than they would be in real‐life settings (i.e., make them artificially similar). Such artificial similarities between arms can lead to spurious conclusions of non‐inferiority. Therefore, if trial‐specific intercurrent events are likely, the estimand must account for them to avoid such spurious conclusion. A *hypothetical* strategy, which considers what outcomes would be if the trial‐specific intercurrent event had *not* occurred, is a good way to do this. However, it is not always possible to distinguish between intercurrent events that would vs. would not occur outside the trial setting. Therefore, the choice of estimand in non‐inferiority trials will depend both on whether trial‐specific intercurrent events are likely, and whether they can be identified (see Table 2).

Abbreviations: ITT = intention to treat, NI = non‐inferiority.

Rather, the threat to validity comes from “trial‐specific” intercurrent events, which we define as intercurrent events that occur in a trial setting but would not occur in routine clinical practice (Box 2). Such trial‐specific intercurrent events may be due to poor study conduct, but may also occur for reasons beyond the investigators' control. For instance, at the start of the COVID‐19 pandemic, many trials faced widespread treatment deviations due to lockdowns or lack of availability of study treatments. Though these deviations reflected usual practice at the time of the trial, they would not be expected to occur to such an extent in the future, and thus can be seen to be trial‐specific. Likewise, many trials leave treatment decisions up to the individual clinicians who treat participants. Due to uncertainty over the best choice of treatment at the time of trial initiation, clinicians may deviate more from the protocol during the trial than they would afterwards, once the uncertainty has been addressed.

BOX 2 **Trial‐specific intercurrent events.**

“Trial‐specific” intercurrent events are those which occur in a trial setting but would not in routine practice. These could occur for different reasons, for instance, changes in context or poor trial conduct. Examples include:

- *External disruptions*: events such as COVID‐19 may lead to treatment interruptions during trials which would not occur once the external disruption was over
- *Clinical hesitancy*: in some trials clinical staff may be hesitant to provide or continue treatment due to uncertainty about the treatment's safety or effectiveness, which may no longer be a concern once the trial is complete and the evidence‐base is improved
- *Built in differences*: some trials require different processes of care to those used in routine practice. For instance, some cancer trials allow control patients to switch to the experimental treatment on disease progression, even if the experimental treatment is not available in real life as a second‐line treatment
- *Accidental*: sometimes accidents leading to intercurrent events may occur in the trial that would not occur in routine care. For instance, in clinical areas where patient care switches rapidly between clinicians, one of the clinicians may be unaware the patient is in the trial and thus not deliver the intended treatment

Trial‐specific intercurrent events can make treatment arms artificially similar which can lead to spurious conclusions of non‐inferiority. Thus, these events may require special consideration when choosing an estimand in order to maintain trial validity.

These trial‐specific intercurrent events serve to make treatment arms more similar than they would be in a non‐trial setting, thus increasing the risk of declaring non‐inferiority when the new intervention is in fact worse than standard treatment. Thus, these intercurrent events require careful handling in the estimand definition in order to avoid spurious conclusions of non‐inferiority.

However, a complication is that trial‐specific intercurrent events may not always be identifiable as such. For instance, in some trials it is possible that there will be more deviations during the trial than would be seen afterwards, once the uncertainty around the optimal treatment is resolved. However, given there will always be some level of non‐compliance in practice, it may be impossible to differentiate between deviations which were trial‐specific and those which would have also occurred outside the trial setting.

Our recommended approach to defining estimands in non‐inferiority trials therefore depends both on whether trial‐specific intercurrent events are likely to be an issue, and if so, whether they can be identified. Our recommendations are given in Table 2. Briefly, if trial‐specific intercurrent events are not expected to occur, there is no need to define a strategy for handling them. In this case, we recommend that a single primary estimand be defined based on clinical considerations, as in any other trial, and non‐inferiority be assessed on the basis of this single estimand.

**TABLE 2:** Recommendations for applying the estimands framework to non‐inferiority trials.

Choice of estimand	
If trial‐specific intercurrent events do *not* occur	A single primary estimand should be chosen based on clinical considerations, as in any other trial design. Non‐inferiority should be assessed on the basis of this estimand.
If trial‐specific intercurrent events *do* occur, and *can* be identified	A single primary estimand should be defined which handles trial‐specific intercurrent events using a *hypothetical* strategy, and is otherwise defined based on clinical considerations. Non‐inferiority should be assessed on the basis of this estimand.
If trial‐specific intercurrent events *do* occur, but *cannot* be identified	Two estimands should be defined: One which assumes there are no trial‐specific intercurrent events and is chosen based on clinical considerations; One which uses a hypothetical strategy for any intercurrent events which may be trial‐specific, as a way to protect against spurious conclusions of non‐inferiority. Non‐inferiority should ideally be assessed on the basis of both estimands. However, depending on the setting of the trial, either (or both) could be specified as a primary estimand, with the other being a secondary or supplementary estimand.

Choice of estimator	
	For trials using a hypothetical strategy to handle trial‐specific intercurrent events, an estimator which targets the hypothetical estimand should be chosen (e.g., inverse probability weighting or the *IV(Bayes)* approach) with the choice depending on which assumptions are most plausible for a given trial. The assumptions behind the chosen estimator should be described, along with a discussion around the plausibility of these assumptions, and sensitivity analyses evaluating robustness of results to deviations from such assumptions if appropriate.

If trial‐specific intercurrent events *are* likely to be an issue and *can* be identified, then we recommend a single primary estimand be specified. The strategies to handle non‐trial‐specific intercurrent events should be chosen based on clinical considerations, as above. However, trial‐specific intercurrent events should be handled using a *hypothetical* strategy (where interest lies in what patient outcomes would have been had the trial‐specific intercurrent events not occurred), in order to match the treatment effect that would be observed in routine practice, and to avoid spurious conclusions of non‐inferiority based on artificial trial‐specific intercurrent events.

Finally, if trial‐specific intercurrent events *are* likely to be an issue and *cannot* be identified, then we suggest that two estimands be specified. First, an estimand should be chosen under the assumption there are no trial‐specific intercurrent events (i.e., that all intercurrent events seen in the trial would also have occurred in practice). Strategies to handle each intercurrent event should be based on clinical considerations, as above. A further estimand should also be specified, which uses a hypothetical strategy for any intercurrent events which *may* be trial‐specific. For instance, in the TOPPS example, a hypothetical strategy would be used to handle any transfusion‐related deviations as it is impossible to distinguish which are trial‐specific and which are not. Then, if non‐inferiority is demonstrated on the basis of both estimands, investigators can be sure it is not a spurious conclusion based on trial‐specific intercurrent events.

In this setting, investigators would need to decide whether to specify both estimands as co‐primary, or one as primary and the other as a secondary or supplemental estimand. Specifying co‐primary estimands with a requirement of non‐inferiority for both may be the most robust approach in terms of reassuring relevant stakeholders that results are not affected by trial‐specific intercurrent events (and notably this approach does not pose any multiplicity issues [22]). For instance, in the regulatory setting where trial‐specific intercurrent events are expected to be common, it may be necessary to use co‐primary estimands and demonstrate non‐inferiority for both in order to be sure that regulatory approval is not given on the basis of a spurious result.

However, power considerations may make the requirement that non‐inferiority be demonstrated on the basis of the confidence intervals for both estimands prohibitive, as many methods to estimate a hypothetical strategy have higher variance than methods to estimate other strategies (such as treatment policy). Therefore, in some situations, it may be useful to define the estimand that assumes no trial‐specific intercurrent events as the primary, and the hypothetical one as a secondary/supplemental estimand. This design would require that NI be shown for the primary estimand, while using results from the secondary hypothetical estimand to ensure the non‐inferiority conclusion was not unduly affected by trial‐specific intercurrent events (i.e., that estimates from the two estimands are not too different). This approach may be useful in non‐regulatory settings where few trial‐specific intercurrent events are expected.

Alternatively, if only a handful of trial‐specific intercurrent events are anticipated, then their impact on results may be negligible and specifying a single estimand that assumes no trial‐specific intercurrent events may be sufficient. However, this choice would require justification, particularly around why few trial‐specific intercurrent events were expected. It is not possible to designate a specific threshold for the proportion of trial‐specific intercurrent events that would warrant using multiple (or co‐primary) estimands that would apply to all NI trials, as the impact of such events on results will depend on various factors (such as the effect of the event on the outcome, which may vary depending on the type of event). In such situations, it may be useful to have early discussions with relevant stakeholders (such as regulators if the trial is to be used for regulatory submission) to discuss the most appropriate approach to take.

## 4 Definition of a Hypothetical Estimand

We now turn our attention to estimation, and we begin by defining the hypothetical estimand, using potential outcomes notation. We define it in terms of a trial with two active treatments (new treatment vs. standard treatment) with “non‐compliance” in both arms as the intercurrent event, where compliance is all‐or‐nothing, that is, either participants receive their allocated intervention or they do not, and there is no switching between the active treatments. Here, we consider non‐compliance to be a trial‐specific intercurrent event; we note that the estimand definition below could easily be extended to include other types of non‐trial‐specific intercurrent events which are handled using alternative strategies.

First, let Y represent the observed outcome, Z the treatment allocation (Z=0 if the patient is allocated to the standard treatment group, Z=1 if allocated to new treatment), and C the patient's compliance status for their assigned treatment (C=1 if the patient complied with their assigned treatment, and C=0 if they did not). Thus, participants can receive one of three treatments: standard treatment or new treatment (if they are assigned to that treatment *and* they comply), or no treatment (if assigned to either treatment arm but they do not comply).

Then, $Y^{\left(Z=1\right)}$ denotes the participant's potential outcome if assigned to the new treatment, and $Y^{\left(Z=0\right)}$ their potential outcome if assigned to standard treatment, and $Y^{\left(Z=1,C=1\right)}$ and $Y^{\left(Z=0,C=1\right)}$ denote their potential outcomes under actual receipt of each treatment.

The hypothetical estimand can then be defined as [20, 23, 24]:

(1) $$E\left(Y^{\left(Z=1,C=1\right)}\right)-E\left(Y^{\left(Z=0,C=1\right)}\right)$$

That is, it is the expected difference in potential outcomes between the new versus standard treatment in the hypothetical setting where all participants would comply with their assigned treatment.

## 5 Overview of Estimators for the Hypothetical Estimand

In this section we describe different estimators which could be used to target the hypothetical estimand. We focus on the setting of two treatment arms with all‐or‐nothing compliance in each, and a continuous outcome, but each estimator could be extended to handle other types of intercurrent events (e.g., treatment switching), or to handle interventions where compliance is not all or nothing (for instance, in TOPPS where clinicians could comply with the transfusion policies at some time points but not others). We briefly mention the additional assumptions required for each estimator when they are extended to the situation of time‐varying treatments (such as in TOPPS). Example Stata code is provided in Table S3 in the Supporting Information to implement these estimators when there is ‘all‐or‐nothing’ compliance in each treatment arm.

We first define some additional notation. Let X denote an observed binary baseline covariate, and let U denote an unobserved binary baseline covariate (both X and U are used to define certain estimators in this section, and are also used in the simulation study in Section 6). For convenience, we assume a single binary variable for both X and U, though this could be extended to multiple variables of different types.

Throughout this manuscript we assume that randomization has been properly implemented, so that the treatment arms are *exchangeable*. We also make two key assumptions for all estimators listed below: (a) *consistency*, that is that $Y=Y^{\left(Z=z,C=c\right)}$ if Z=z and C=c, for z=0,1 and c=0,1; and (b) *no interference*, that is that $Y^{\left(Z=z,C=c\right)}$ is independent of the Z and C values of other participants [25].

### 5.1 Intention‐to‐Treat

An intention‐to‐treat approach is generally used to estimate a treatment policy estimand, and is thus typically not appropriate for a hypothetical strategy. However, we include it here for illustrative purposes. In the context of a continuous outcome, this estimator would typically involve applying a linear regression model of the outcome Y on treatment Z to the intention‐to‐treat population, which includes all participants in the trial, regardless of whether they complied or not. Because this approach estimates a treatment policy estimand, it will therefore only be unbiased for the hypothetical estimand when the two coincide. This could occur, for instance, if (i) there is no non‐compliance; or (ii) potential outcomes under compliance are the same as under non‐compliance (i.e., when $Y^{\left(Z=z,C=1\right)}=Y^{\left(Z=z,C=0\right)}$). When neither of these conditions are true, this estimator will typically be biased for the hypothetical estimand.

### 5.2 Per‐Protocol

A per‐protocol analysis is the same as the ITT approach described above, except that participants who did not comply are excluded from the analysis population. Per‐protocol analyses can adjust for baseline covariates, such as X, as a covariate in a regression model, in case such covariates act as confounders between the outcome and non‐compliance.

For “all‐or‐nothing” treatments, the assumptions required for unbiasedness are:

- *Conditional exchangeability*, that is that $Y^{\left(Z=z,C=1\right)}⊥Z,C\;|X$. This implies that, conditional on X, patients who comply with their assigned treatment are *exchangeable* between treatment arms. This is sometimes referred to as the “no unmeasured confounding” assumption [23].
- The association between X and outcome Y has been correctly specified (i.e., that there is no residual confounding due to misspecification of the confounder‐outcome association).
- No treatment effect heterogeneity across levels of X.

The last assumption is required because the per‐protocol analysis provides a weighted average of the estimated treatment effects across levels of X, however the weighting used does not necessarily correspond to population weights for X. Thus, if the treatment effect varies across levels of X, then the per‐protocol analysis may upweight or down weight treatment effects from certain levels of X more than it should.

It should be noted that for non‐collapsible summary measures, such as an odds ratio, adjustment for baseline covariates can change the estimand from a marginal one to a conditional one, which may not be desirable. However, this is not an issue for differences, and so we do not consider this issue further here.

For time‐varying treatments or those with partial compliance (where compliance is defined as meeting some threshold of treatment adherence), per‐protocol analyses do not require any additional assumptions for unbiasedness, but the plausibility of the “no unmeasured confounding” assumption becomes much less likely because it needs to hold at each time point, and there may be post‐randomization confounding factors which cannot be adjusted for in the analysis.

### 5.3 Inverse Probability Weighting

For “all‐or‐nothing” interventions, inverse probability weighting (also termed “inverse probability of censoring weighting”) estimates hypothetical treatment effects by excluding participants who did not comply, and re‐weighting participants who did comply according to the inverse of their probability of complying [23, 26, 27, 28, 29]. Broadly, the idea is to implicitly impute what outcome data for participants who did not comply would have been under hypothetical compliance, by up‐weighting outcome data from comparable participants who did comply.

IPW is implemented in two stages. The first stage is used to estimate the weights to be used in the second stage. This is done separately within each treatment arm, and the weights are defined as:

$$W_{Z=0}=\frac{1}{\hat{P}\left(C=1|Z=0,X\right)}$$

and:

$$W_{Z=1}=\frac{1}{\hat{P}\left(C=1|Z=1,X\right)}$$

where $\hat{P}\left(C=1|Z=0,X\right)$ and $\hat{P}\left(C=1|Z=1,X\right)$ are estimated using a logistic regression model applied to each arm separately with the participant's compliance status as the outcome, and baseline covariate(s) X as covariates. Then, $\hat{P}\left(C=1|Z=0,X\right)$ and $\hat{P}\left(C=1|Z=1,X\right)$ are the participant‐specific predictions from the logistic models.

In the second stage, the treatment effect is estimated using a weighted regression model, with outcome Y, treatment allocation Z, and weights $W_{Z=0}$ (if Z=0) and $W_{Z=1}$ (if Z=1).

For “all‐or‐nothing” treatments, the assumptions required for unbiasedness are [23, 26, 27, 28, 29]:

- *Conditional exchangeability*, that is, that all the relevant X variables have been used to estimate the weights, $W_{Z=0}$ and $W_{Z=1}$, so that $Y^{\left(Z=z,C=c\right)}⊥Z,C|X$
- The association between X and compliance status C has been correctly specified to estimate the weights during stage 1 (i.e., that there is no residual confounding due to misspecification of the confounder‐compliance association)
- There is a non‐zero probability of complying in each treatment arm for all combinations of the baseline covariates (this is known as the “positivity” assumption)

For time‐varying treatments, the IPW approach described above must be extended to deal with time‐varying confounding between post‐randomization variables and compliance status (e.g., if post‐randomization blood measurements make non‐compliance more likely) [26, 27, 28, 29]. This is done by splitting the follow‐up period into distinct time‐points, and calculating weights for each distinct time‐point (based on the inverse probability of remaining compliant at that time‐point, conditional on the participant being compliant up to that point); calculation of these weights would include post‐randomization confounders of compliance status and outcomes at each follow‐up time‐point. IPW does not require any additional assumptions for unbiasedness in this setting, except that the assumptions listed above now include post‐randomization confounding and positivity (i.e., all baseline *and* post‐randomization confounders have been included and correctly modeled, and there is a non‐zero probability of remaining compliant at all follow‐up time‐points for all combinations of covariates given the compliance history). For time‐varying treatments, weights may need to be stabilized [29].

### 5.4 Instrumental Variables

Instrumental variables (IV) is an analysis technique which uses “instruments” to estimate the effect of adhering to treatment [30, 31, 32, 33, 34]. An instrument is a variable that is associated with compliance, but not associated with the outcome *except* through its impact on compliance [30, 31, 33]. A major benefit of IV methods is that they do not require the “no unmeasured confounding” assumption, and thus can provide unbiased estimates even when confounding between compliance status and the outcome occurs. However, they make alternative assumptions which may be more or less plausible depending on context.

We define some additional notation. Let $C_{0}$ denote actual receipt of treatment 0, so $C_{0}=1$ if Z=0 and C=1, and 0 otherwise, and $C_{1}$ denote actual receipt of treatment 1, so $C_{1}=1$ if Z=1 and C=1, and 0 otherwise. In randomized trials, randomized arm (Z) is typically used as an instrument, though as discussed below, some estimators require additional instruments. There are three essential requirements for a variable to be a valid instrument [30, 31, 33] (and further assumptions for an estimator based on IVs to be unbiased for the hypothetical estimand which are discussed below):

1. The instrument must be associated with treatment actually received (e.g., randomization to treatment Z=1 is associated with patients actually receiving treatment 1, denoted by $C_{1}$);
2. The instrument has no effect on the outcome Y except through its effect on treatment received, $C_{0}$ and $C_{1}$ (this is commonly referred to as the “exclusion restriction” and means that treatment allocation Z does not causally affect outcome Y in participants for whom C=0);
3. The instrument does not share any common causes with the outcome Y (i.e., the association between Z and Y is unconfounded).

Using randomized arm, Z, as an instrument typically fulfills assumptions 1 and 3, though the plausibility of assumption 2 requires context‐specific knowledge (e.g., it is plausible for many all‐or‐nothing treatments, but perhaps less plausible for interventions with partial compliance where $C_{0}$ and $C_{1}$ are defined as fully adhering to treatment) [33].

One additional assumption required to estimate the hypothetical estimand is *homogeneity*, i.e. that the treatment effect under hypothetical compliance is the same across all compliance levels. Broadly, this implies the quantity $E\left(Y^{\left(Z=1,C=1\right)}-Y^{\left(Z=1,C=1\right)}\right)$ is identical for patients who would comply under either treatment assignment; for those who would comply under assignment to one treatment but not the other; or for those who would not comply under assignment to either treatment.

IV estimation can be best explained using a two‐stage approach. Without covariates, the two stages are [33]:

1. Stage 1: a linear regression model is fitted for each treatment arm, with receipt of treatment ($C_{0}$ or $C_{1}$) as the outcome and allocation (Z) as the covariate (so that participants not assigned to treatment Z=0 are included in the model as $C_{0}=0$, and similarly for participants not assigned to Z=1). A prediction for each participant's treatment received status is then obtained (${\hat{C}}_{0}$ and ${\hat{C}}_{1}$)
2. Stage 2: a linear regression model is fitted with Y as the outcome, and compliance predictions ${\hat{C}}_{0}$ and ${\hat{C}}_{1}$ as covariates. An overall estimate of treatment effect is then obtained by contrasting the estimated parameters for ${\hat{C}}_{1}$ and ${\hat{C}}_{0}$

A challenge for trials with non‐compliance in both treatment arms is that, without covariates, ${\hat{C}}_{1}$ and ${\hat{C}}_{0}$ are necessarily collinear, leading to an unidentifiable model in stage 2. Thus, IV methods for trials with non‐compliance in both treatments require additional assumptions to resolve the collinearity issue. We discuss two of these estimators below.

#### 5.4.1 IV Method 1: IV(Interaction)

Fischer et al. [35] described an IV approach which avoids collinearity between ${\hat{C}}_{1}$ and ${\hat{C}}_{0}$ by using randomized arm (Z) as the first instrument, and then specifying a second instrument based on the interaction between treatment allocation and a baseline covariate (ZX). This approach has also been discussed by others [33, 36]. We refer to this approach as *IV(interaction)*. In the present setting, including covariates in the stage 1 model naturally leads to the use of interactions, because $C_{1}$ is identically zero in Z=0 but may vary with X in Z=1 (and vice versa).

The stage 1 models for this approach are:

(2) $$C_{0}=\left\{\begin{array}{ll} \alpha^{C0}+\beta_{X}^{C0}X+e^{C0} & \text{if}\;Z=0\\ 0 & \text{if}\;Z=1 \end{array}\right.$$

(3) $$C_{1}=\left\{\begin{array}{ll} 0 & \text{if}\;Z=0\\ \alpha^{C1}+\beta_{X}^{C1}X+e^{C1} & \text{if}\;Z=1 \end{array}\right.$$

and the stage 2 model is:

(4) $$Y=\alpha^{Y}+\beta_{C0}^{Y}{\hat{C}}_{0}+\beta_{C1}^{Y}{\hat{C}}_{1}+\beta_{X}^{Y}X+e^{Y}$$

where $e^{Y}$, $e^{C0}$, and $e^{C1}$ are residual error terms, assumed to be normally distributed with mean zero and (co‐)variances specified by a 3 × 3 matrix. We use superscripts Y, $C_{0}$, and *C*_1_ to denote which model each parameter belongs to.

The treatment effect is then estimated as ${\hat{\beta }}_{C1}^{Y}-{\hat{\beta }}_{C0}^{Y}$ from model (4).

The key idea behind this estimator can be summarized as follows (for full details, see Fischer et al. [35]): Z and ZX are used as instruments, and for these to be valid instruments they must predict compliance in both treatment arms (the variables $C_{0}$ and $C_{1}$). Z predicts both $C_{0}$ and $C_{1}$, provided there is some compliance in both treatment groups (i.e., E[C|Z]>0 for Z=0,1); and ZX predicts $C_{1}$ if X predicts compliance within treatment arm Z=1. Model (4) is identifiable provided the covariates are not collinear: Fischer et al. showed that this is true provided the predicted treatment compliances ${\hat{C}}_{0}$ and ${\hat{C}}_{1}$ are not proportional across levels of the baseline covariate, that is, there is no k such that ${\hat{C}}_{1}=k{\hat{C}}_{0}$ across all levels of X. A further requirement for the hypothetical estimand is that the baseline covariate X does not moderate the treatment effect directly (i.e., there is no baseline‐by‐treatment interaction on outcome) [33].

This estimator could be used for time‐varying treatments where compliance is defined as meeting some adherence threshold (e.g., compliant for 80% of study days), however the exclusion restriction assumption above (that Z is not associated with outcome Y in participants for whom C=0) is likely to be violated. For instance, in TOPPS it is likely that a patient who followed the transfusion protocol for 28/30 platelet counts is going to receive some benefit from being allocated to that treatment arm.

#### 5.4.2 IV Method 2: IV(Bayes)

For many non‐inferiority trials, information on the effect of standard vs. no treatment will be available from previous trials which have compared the standard treatment against placebo or previous controls. Bond and White [37] therefore described an IV approach which handles collinearity between ${\hat{C}}_{1}$ and ${\hat{C}}_{0}$ by using a Bayesian framework to put an informative prior on the parameter $\beta_{C0}^{Y}$ (the effect of the standard treatment vs. no treatment) in the stage 2 model. Even if previous trial information is not available, it may still be possible to identify plausible priors for $\beta_{C0}^{Y}$, for instance based on clinical knowledge. Other parameters use uninformative priors (though could be made informative if desired). We refer to this approach as *IV(Bayes)*.

The stage 1 models for this approach, without adjustment for covariates, are:

(5) $$C_{0}=\alpha^{C0}+\beta_{Z}^{C0}Z+e^{C0}$$

(6) $$C_{1}=\alpha^{C1}+\beta_{Z}^{C1}Z+e^{C1}$$

and the stage 2 model is:

(7) $$Y=\alpha^{Y}+\beta_{C0}^{Y}{\hat{C}}_{0}+\beta_{C1}^{Y}{\hat{C}}_{1}+e^{Y}$$

where an informative prior is placed on $\beta_{C0}^{Y}$ in model (7), and uninformative priors on other parameters in the model. The treatment effect is then estimated as ${\hat{\beta }}_{C1}^{Y}-{\hat{\beta }}_{C0}^{Y}$.

Although this is a Bayesian approach, we still discuss the assumptions required for this estimator to be unbiased for the hypothetical estimand. For a given prior placed on $\beta_{C0}^{Y}$, an unbiased treatment effect also requires the mean of the prior to be an unbiased representation of the true effect.

Similarly to the *IV(interaction)* method, this approach could be used for time‐varying treatments however violations to the exclusion restriction are more likely, which may introduce bias.

## 6 Simulation Study Methods

We conducted a simulation study to evaluate the estimators described earlier. The primary aim was to evaluate bias both when the estimators' assumptions were fulfilled, as well as when the assumptions were violated. Secondary aims were to evaluate precision and type I error rate of the estimators.

Simulations were based on a two‐arm randomized non‐inferiority trial with a continuous outcome. Non‐compliance occurred in both treatment arms, and compliance was “all or nothing,” that is, patients either received their allocated treatment or received nothing.

We performed two simulations studies: the first when there is no treatment effect heterogeneity across compliance levels (a core assumption of the IV methods and the per‐protocol analysis), and the second which did include treatment effect heterogeneity across compliance levels (indicating a violation in assumptions for the IV and per‐protocol estimators).

Full details of the simulation methods, including exact parameter values for all scenarios, are available in the Supporting Information. Stata code used to generate and analyze data is available in the supplementary do‐file for two scenarios (one each for simulation studies 1 and 2), and code for the other scenarios was identical except for modifications to the input parameters. Below we summarize the key aspects of the simulation study.

### 6.1 Simulation Study 1 (No Treatment Effect Heterogeneity)

We generated patient outcomes in two steps. First, we generated whether they complied with their assigned treatment, and then we generated their outcome. Their compliance could depend on treatment allocation Z, an observed baseline covariate X, and unobserved baseline covariate U, and the interactions between treatment allocation and either the observed or unobserved baseline covariate. Their outcome could depend on treatment received, and the observed and unobserved baseline covariates. Inclusion of an interaction between Z and X in the model to generate compliance was used to generate measured confounding between compliance status and outcome, while inclusion of an interaction between Z and U was used to generate unmeasured confounding (see Table 3).

**TABLE 3:** Summary of compliance scenarios for simulation study 1.

Scenario	Compliance type	Is association between X/U and compliance the same or different between treatment arms?	Description
1	Compliance does not depend on any observed or unobserved baseline covariates	Same	The probability of compliance is the same for all patients
2a	Compliance depends only on observed baseline covariates (X)	Same	Healthier patients are less likely to comply for both treatments, and there is higher compliance to the new treatment
2b		Different	Healthier patients are less likely to comply to the standard treatment, but more likely to comply to the new treatment
2c		Different	Healthier patients are less likely to comply to the standard treatment, but more likely to comply to the new treatment, and there is higher compliance to the new treatment
3a	Compliance depends on both observed and unobserved baseline covariates (X and U)	Same	Healthier patients are less likely to comply for both treatments, and there is higher compliance to the new treatment
3b		Different	Healthier patients are less likely to comply to the standard treatment, but more likely to comply to the new treatment
4a	Compliance depends only on unobserved baseline covariates (U)	Same	Healthier patients are less likely to comply for both treatments, and there is higher compliance to the new treatment
4b		Different	Healthier patients are less likely to comply to the standard treatment, but more likely to comply to the new treatment

We considered five scenarios (labeled A–E, shown in Table S4) in which we varied the sample size, percentage compliance, true value of the estimand, and association between covariates X and U and outcome. Then, for each of these five scenarios, we also considered eight compliance scenarios (labeled 1, 2a–c, 3a,b, and 4a,b; shown in Tables 3 and S4). This led to a total of 5 × 8 = 40 scenarios. We used a non‐inferiority margin of −0.3 in all scenarios.

The aims of scenarios A–E were to assess the impact of smaller versus larger sample sizes, smaller versus larger degrees of non‐compliance, smaller versus larger associations between covariates and the outcome, as well as impacts on type I error rate versus power. The aims of compliance scenarios 1–4 were to evaluate the impact of different types of compliance mechanisms, including when there was no measured or unmeasured confounding between compliance and outcome, when there was measured confounding only, unmeasured confounding only, or both.

### 6.2 Simulation Study 2 (Treatment Effect Heterogeneity)

We used two treatment effect heterogeneity (TEH) scenarios: one in which the treatment effect varied across values of X (which implies it varies across compliance status, as X is strongly associated with compliance in this scenario), and one in which it varied across values of U (which also implies it varies across compliance status; the key difference between these scenarios is that X is observed while U is not). We label these two scenarios *TEH(X) and TEH(U)*.

For *TEH(X)* and *TEH(U)* we generated data so that there was observed and unobserved confounding between compliance and outcome respectively. For both scenarios we varied two factors: the degree of TEH (moderate vs. large), and the difference in compliance between treatment groups (moderate vs. large).

The aim of simulation study 2 was to evaluate how estimators performed when there was treatment effect heterogeneity as well as confounding between compliance status and outcome (either observed or unobserved).

### 6.3 Estimators

We implemented five estimators, as described earlier; (i) intention‐to‐treat; (ii) per‐protocol; (iii) IPW; (iv) *IV(Bayes)*; and (v) *IV(interaction)*. All analyses adjusted for the observed covariate X (except for IPW, which used X to estimate weights). Our primary interest was in evaluating IPW, *IV(Bayes)*, and *IV(interaction)*, however we included intention‐to‐treat and per‐protocol for completeness. We note that, as discussed earlier, ITT targets a treatment policy strategy, and should not be used when a hypothetical strategy is of interest. However, we have included it here for two reasons. First, to demonstrate the impact on results when trial‐specific intercurrent events are ignored, and second, to empirically demonstrate the variance inflation of the other estimators, which has implications for the trial's design.

For *IV(Bayes)*, we evaluated four different priors for the effect of standard treatment versus no treatment: (a) a well centered, precise prior (i.e., where the prior's mean matches the true mean in the trial, and the prior has a small variance); (b) a well centered, vague prior (where the prior has a large variance); (c) a miscentred, precise prior (where the prior's mean does not match the true mean in the trial); and (d) a miscentred, vague prior. We used these four priors to evaluate the impact of misspecifying the prior's mean in relation to the true mean in the trial, as well as the impact of more versus less precise priors.

We evaluated estimators based on frequentist properties for bias (our main objective), as well as precision, type I error rate, and bias in estimated standard errors (our secondary objectives).

For each estimator, standard errors were calculated using the default approach in Stata. The estimators that use Stata's regress command (intention‐to‐treat and per‐protocol) used ordinary least squares estimates of standard errors. The IPW method was implemented using a weighted version of the regress command, and the standard errors were obtained from a sandwich estimator. The IV(Bayes) method uses the standard deviation of the posterior distribution as the standard error of the estimator, and the lower end of the 95% credible interval to determine whether non‐inferiority is declared or not. The IV(interaction) estimator uses the default variance estimator given in the Stata manual for ivregress [38].

## 7 Simulation Study Results

### 7.1 Simulation Study 1 (No Treatment Effect Heterogeneity)

Full results for each simulation scenario are available in the Supporting Information. Because results between scenarios A‐E were broadly similar, we present the results for scenario A below. Our focus is on describing results for IPW, *IV(Bayes)*, and *IV(interaction)*, however intention‐to‐treat and per‐protocol results are available in the figures and in the Supporting Information.

#### 7.1.1 Bias in Estimated Treatment Effects

**FIGURE 1:**
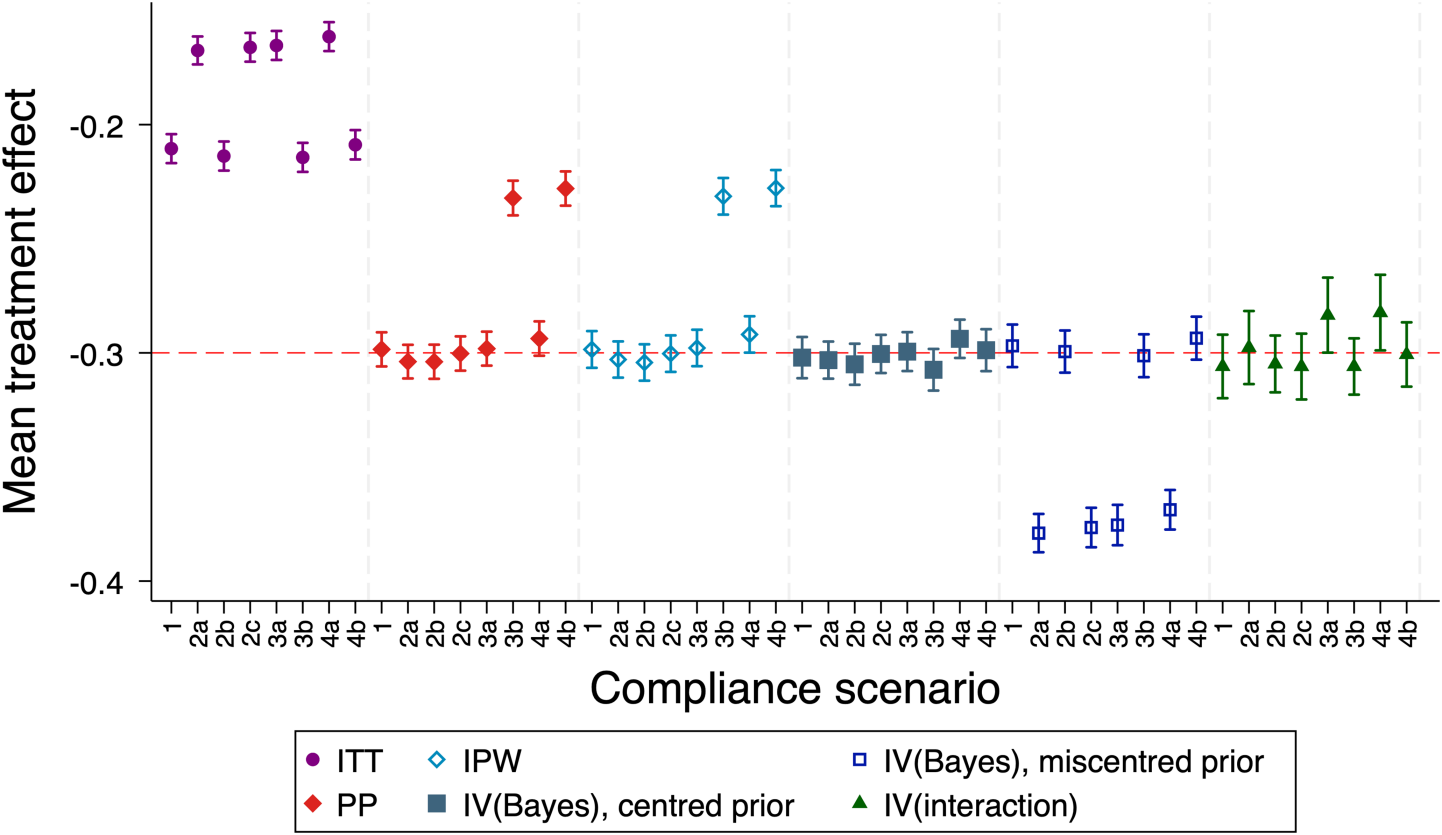
Mean estimates of treatment effect for simulation study 1 (no treatment effect heterogeneity), scenario A (true value −0.3, trial sample size 100, overall compliance level 70%). IPW = inverse probability weighting, ITT = intention‐to‐treat, IV = instrumental variables. PP = per‐protocol. Note that the ITT estimator is targeting a different estimand to the other estimators (treatment policy), with a different true value of the estimand, and is included here for illustrative purposes only. *IV(interaction)* results are reported for the subset of replications where the SE was ≤ 10 times the empirical SE from ITT. IPW is reported for the subset of replications for which no observations were dropped due to perfect prediction in the logistic regression generating weights. *IV(Bayes)* results are presented for the precise prior.

Results are shown in Figure 1 and the Supporting Information. As expected, IPW was unbiased except when there was unmeasured confounding between compliance status and outcome. *IV(Bayes)* was unbiased except when the mean of the prior for the effect of the standard treatment vs. no treatment was mispecified compared to the true mean in the trial. However, when the overall compliance rate was the same in each treatment group, *IV(Bayes)* was unbiased even when the prior was mispecified.

*IV(interaction)* was extremely unstable across all scenarios (see Supporting Information), and results were severely affected by extreme outliers. After removing replications with extreme values, the method performed better, though was still biased for certain scenarios.

#### 7.1.2 Precision and Type I Error Rate

**FIGURE 2:**
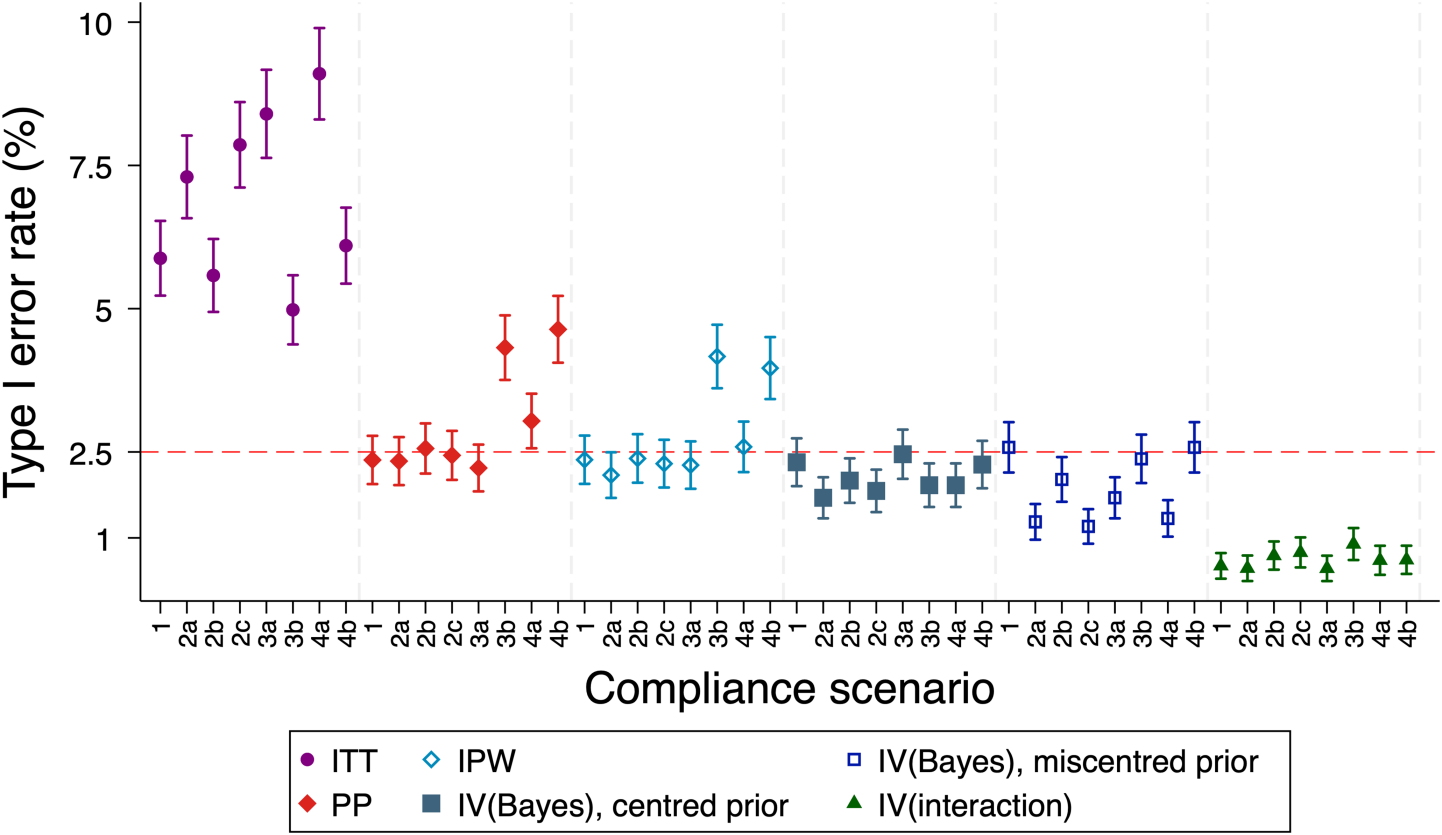
Type I error rate for simulation study 1 (no treatment effect heterogeneity), scenario A (nominal value set at 2.5%, trial sample size 100, overall compliance level 70%). IPW = inverse probability weighting, ITT = intention‐to‐treat, IV = instrumental variables, PP = per‐protocol. Note that the ITT estimator is targeting a different estimand to the other estimators (treatment policy), with a different true value of the estimand, and is included here for illustrative purposes only. *IV(interaction)* results are reported for the subset of replications where the SE was ≤ 10 times the empirical SE from ITT. IPW is reported for the subset of replications for which no observations were dropped due to perfect prediction in the logistic regression generating weights. *IV(Bayes)* results are presented for the precise prior.

**FIGURE 3:**
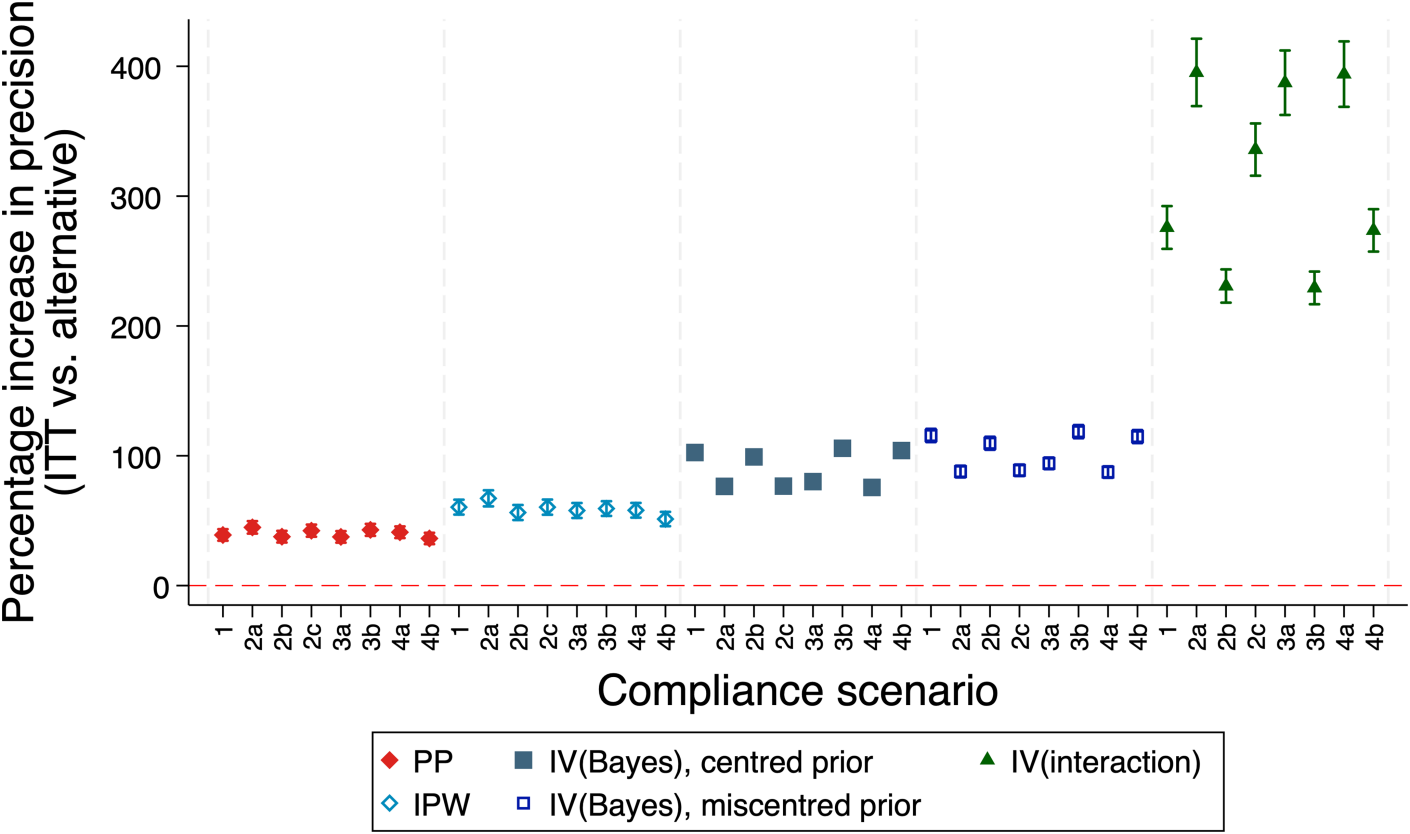
Percentage increase in precision of ITT estimator versus other estimators for simulation study 1 (no treatment effect heterogeneity), scenario A (trial sample size 100, overall compliance level 70%). Defined as $100\times \left({\left(\frac{{\mathrm{SE}}_{\text{alternative}}}{{\mathrm{SE}}_{\mathrm{ITT}}}\right)}^{2}-1\right)$ where $SE_{\text{method}}$ is the empirical standard error. Values > 0 denote ITT is more precise than the comparator method. IPW = inverse probability weighting, ITT = intention‐to‐treat, IV = instrumental variables, PP = per‐protocol. *IV(interaction)* results are reported for the subset of replications where the SE was ≤ 10 times the empirical SE from ITT. IPW is reported for the subset of replications for which no observations were dropped due to perfect prediction in the logistic regression generating weights. *IV(Bayes)* results are presented for the precise prior.

Results are shown in Figures 2 and 3, and the Supporting Information. As expected, IPW led to inflated type I error rates in the same scenarios for which it was biased (i.e., when there was unmeasured confounding), but maintained type I error rates otherwise. In some simulated datasets, the IPW method dropped some observations due to perfect prediction in the logistic regressions used to generate weights. This generally affected only a small number of datasets in each scenario (between 0% and 0.8% for most scenarios; see tables in Supporting Information) but was as high as 4.1% when compliance was particularly high.

*IV(Bayes)* controlled the type I error rate at close to the nominal level when the mean of the prior was well specified, but resulted in some type I error rates which were too low when the prior was mispecified. This was because treatment effect estimates were biased away from the null, which made a finding of non‐inferiority less likely.

*IV(interaction)* led to type I error rates that were far below the nominal level for all scenarios. This was primarily due to extreme bias in estimated SEs.

In general, IPW was more precise than *IV(Bayes)*, and *IV(interaction)* was the least precise, with losses in precision up to 400% in some cases.

#### 7.1.3 Precise Versus Vague Priors for *IV(Bayes)* Approaches

Full results are available in the Supporting Information. Using a precise versus vague prior had no impact on bias in estimated treatment effects. The precision of the two approaches were very similar, however the estimated SEs from the vague prior were substantially biased upwards (often > 20%), which led to type I error rates that were below the nominal level in many cases, and reduced power. Overall, we did not find any benefit in frequentist properties to using a vague prior over a precise prior.

### 7.2 Simulation Study 2 (Treatment Effect Heterogeneity)

**FIGURE 4:**
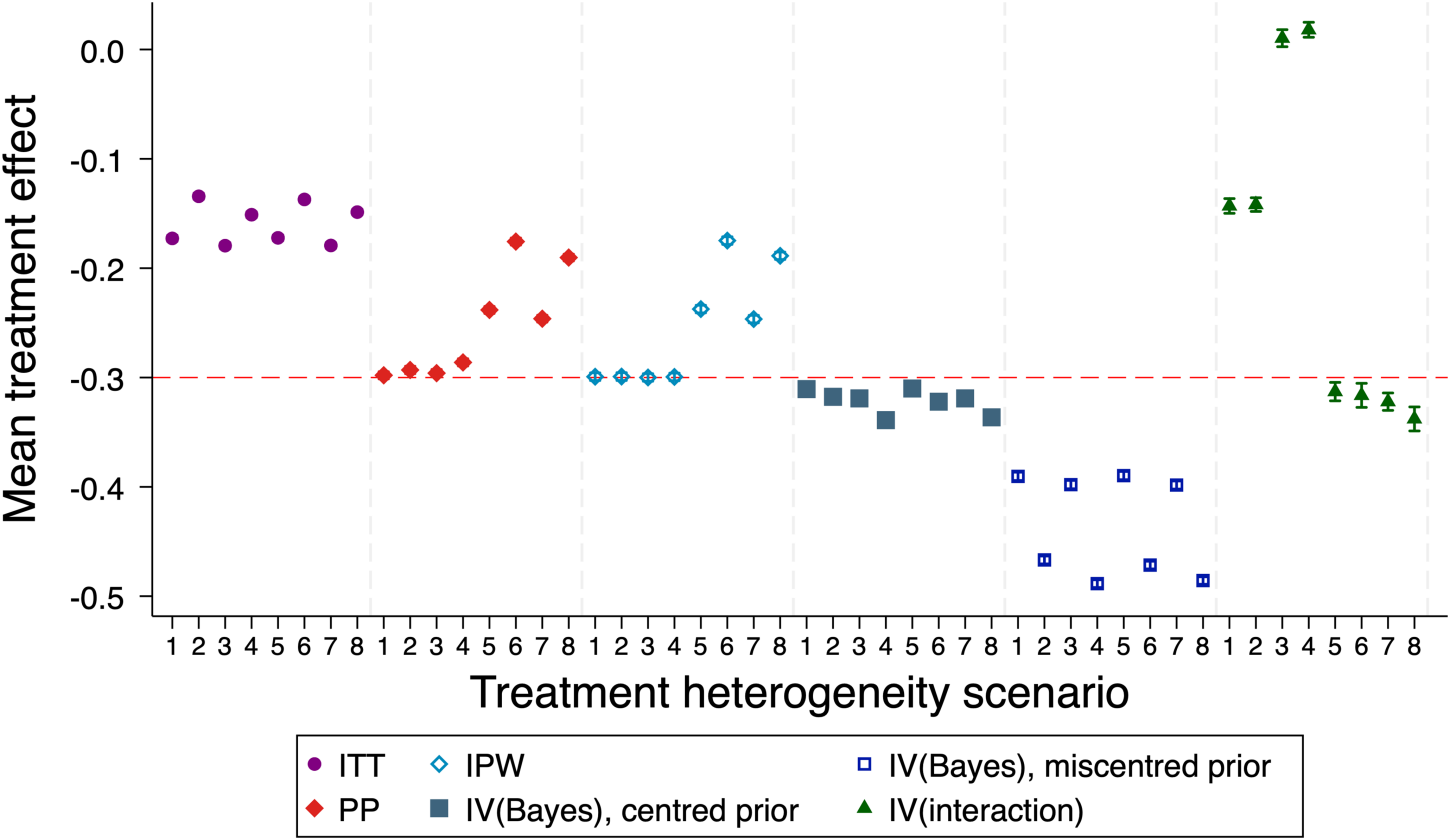
Mean estimates of treatment effect for simulation study 2 (treatment effect heterogeneity) (true value −0.3, trial sample size 500, overall compliance level 70%). IPW = inverse probability weighting, ITT = intention‐to‐treat, IV = instrumental variables, PP = per‐protocol. Scenarios 1–4 relate to TEH across X (an observed baseline covariate), while scenarios 5–8 relate to TEH across U (an unobserved baseline covariate). Scenario 1 contains moderate compliance differences across treatment arms, and moderate TEH; scenario 2 contains large compliance differences and moderate TEH; scenario 3 contains moderate compliance differences and large TEH; and scenario 4 contains large compliance differences and large TEH. A similar pattern occurs for scenarios 5–8. Note that the ITT estimator is targeting a different estimand to the other estimators (treatment policy), with a different true value of the estimand, and is included here for illustrative purposes only. *IV(interaction)* results are reported for the subset of replications where the SE was ≤ 10 times the empirical SE from ITT. IPW is reported for the subset of replications for which no observations were dropped due to perfect prediction in the logistic regression generating weights. IV(Bayes) results are presented for the precise prior.

Mean estimated treatment effects are shown in Figure 4. IPW was unbiased in scenarios where there was no unmeasured confounding. *IV(Bayes)* with a centered prior had a slight bias in all scenarios, which was more pronounced when there was both a large degree of TEH and large differences in compliance between treatment arms. *IV(Bayes)* with a miscentred prior was extremely biased across all scenarios, as was *IV(interaction)* for most scenarios.

## 8 Re‐Analysis of TOPPS Trial

### 8.1 Methods

We re‐analyzed the TOPPS trial to compare the different estimators in practice. We analyzed a secondary outcome, the number of days with bleeding. We chose to analyze this instead of the primary outcome described in Section 2 because we wanted to compare the analysis methods on a continuous outcome to match our simulation study. Further, this outcome displays similar results to the primary outcome, where the per‐protocol analysis led to a smaller estimate of treatment effect than the intention‐to‐treat analysis.

A full description of methods is available in the Supporting Information. Briefly, for intention‐to‐treat and per‐protocol we adjusted for baseline variables which we thought might act as potential confounders (i.e., be associated with both the outcome and deviation from the assigned transfusion strategy). These were: relapsed disease, previous stem cell transplantation, fungal infection, and organ failure.

For IPW, we analyzed each study day separately, to allow for the fact that deviations from assigned transfusion strategy could occur at any point during the study period. We excluded data after the first deviation, then calculated weights using two post‐randomization variables (whether the patient had previously experienced a minor bleed during follow‐up [WHO grade 1], and whether the patient had previously experienced a major bleed during follow‐up [WHO grade 2–4]). The model further adjusted for the same baseline covariates as the per‐protocol and intention‐to‐treat analyses.

For *IV(interaction)* we fitted four separate models, each using one of the baseline covariates described above (relapsed disease, previous stem cell transplantation, fungal infection, and organ failure) as an instrument; the interaction between each covariate and treatment allocation on compliance is shown in the Supporting Information (Table S6).

For the *IV(Bayes)* approach we fitted four separate models, each using a different prior for the effect of the active control (the prophylactic strategy). In our earlier descriptions of this method, priors were based on the effect of the standard treatment vs. no treatment, reflecting the setting where patients who deviate do not receive any treatment. However, this is not the case in TOPPS, and so the priors need to reflect the effect of the prophylaxis strategy vs. the treatment patients who deviate from the prophylaxis strategy would receive: the effect of prophylaxis vs. receiving a transfusion at a higher platelet count than the prophylaxis strategy calls for. Priors were chosen based on our judgment of what was plausible; this was done specifically for the purpose of this re‐analysis, and so they were chosen retrospectively after the trial was already complete. For the four priors, we used combinations of small vs. large effects and precise versus vague variances. We chose an increase of 2 days with bleeding as a large effect, on the basis that following the prophylaxis strategy leads to lower platelet counts than deviating from it (i.e., transfusing before the platelet count dropped below the prophylaxis threshold), and lower platelet counts may increase the risk of bleeding. We chose no difference in days with bleeding as a small effect, under the assumption that as long as the platelet count is above the prophylaxis threshold, there is little difference in the risk of bleeding. We used precise and vague variances of 1 and 10 respectively.

Of note, all of these analyses make strong assumptions that cannot be verified using the trial data. For instance, IPW assumes all confounders between bleeding and transfusion deviations were measured and included in the weighting model with the correct functional form. *IV(interaction)* requires several assumptions, including an interaction between the covariate and treatment on compliance; no treatment‐by‐covariate interaction on outcome; and that patients with transfusion deviations receive no benefit from the assigned strategy. Finally, *IV(Bayes)* requires that the chosen prior is well centered on the true effect of the prophylactic strategy vs. receiving a transfusion at a higher platelet count.

### 8.2 Results

Results are shown in Table 4. For the number of days with bleeding, the ITT and per‐protocol analyses had discrepant results; ITT showed a statistically significant increase (difference non‐prophylactic vs. prophylactic 0.6 days, 95% CI 0.2 to 1.0, *p* = 0.004) while per‐protocol did not (difference 0.4 days, 95% −0.1 to 0.8, *p* = 0.11). IPW and *IV(Bayes)* also demonstrated significant increases in bleeding days; IPW and *IV(Bayes)* with a small prior both showed results similar to ITT, while *IV(Bayes)* with a large prior showed a larger increase in bleeding days (1.2, 95% CI 0.7 to 1.7). Results for *IV(interaction)* were highly variable depending on which baseline covariate was used as the basis for an instrument; estimates ranged between −1.2 and 3.9. One covariate gave an estimate in the opposite direction as the ITT and per‐protocol results, another indicated no effect, and one gave an estimate that was about 6.5 times larger than the ITT effect.

**TABLE 4:** Results from re‐analysis of TOPPS trial.

	Number of days with bleeding	
	Estimated difference in means (95% CI^a^)	*p*
ITT	0.6 (0.2 to 1.0)	0.004
PP	0.4 (−0.1 to 0.8)	0.11
IPW	0.7 (0.2 to 1.1)	0.005
IV(interaction)^b^		
Relapsed disease	−1.2 (−9.7 to 7.4)	0.79
Previous SCT	0.0 (−15.9 to 15.9)	> 0.99
Fungal infection	3.9 (−10.2 to 17.9)	0.59
Organ failure	1.1 (−0.1 to 2.3)	0.07
IV(Bayes)^c^		
Large effect, precise	0.5 (0.0 to 0.9)	—
Small effect, precise	0.7 (0.2 to 1.2)	—
Large effect, vague	0.5 (−0.3 to 1.3)	—
Small effect, vague	0.7 (−0.1 to 1.5)	—

Abbreviations: IPW = inverse probability weighting, ITT = intention‐to‐treat, IV = instrumental variables, PP = per‐protocol.

^a^ CI = confidence interval for ITT, PP, IPW, IV; credible interval for Bayes.

^b^ Baseline characteristics used as instruments.

^c^ Priors were for effect of prophylaxis versus receiving a platelet transfusion against protocol (i.e., at a higher threshold than the prophylaxis strategy calls for): large/precise˜*N*(1, 2), small/precise˜*N*(0, 1), large/vague˜*N*(2, 10), and small/vague˜*N*(0, 10).

## 9 Application of Our Framework to a Prospective Trial Comparing Different Platelet Transfusion Strategies

We now demonstrate how our proposed framework could be applied to a hypothetical trial comparing platelet transfusion strategies based on different thresholds, e.g., a trial similar to TOPPS but comparing a restrictive transfusion threshold (e.g., 7 × 10^9^ per liter) with a liberal transfusion threshold that is standard of care (e.g., 10 × 10^9^ per liter), where patients are given a platelet transfusion whenever their platelet count drops below the assigned threshold. If the restrictive threshold were to be non‐inferior, it would reduce the risk of transfusion related adverse events and offer cost and resource savings.

Considerations for choice of estimand and estimator are shown in Box 3. Briefly, trial‐specific intercurrent events (where clinicians deviate from the assigned transfusion strategy in the trial where they would not in practice, if the restrictive approach was shown to be non‐inferior) are thought to be likely, but would not be distinguishable from non‐trial‐specific intercurrent events.

BOX 3 **Application of the framework to a prospective trial comparing a restrictive and a liberal platelet transfusion threshold.**

*Whether trial‐specific intercurrent events are likely and can be identified*

Investigators decide that trial‐specific intercurrent events are most likely to occur if clinicians deviate from the restrictive transfusion threshold in the trial over concerns around its safety, but would deviate less in routine clinical practice if the restrictive threshold were found to be safe (i.e., non‐inferior).

Though impossible to know for sure, investigators anticipate this type of trial‐specific intercurrent event is likely to occur, but not frequently (e.g., affecting around 5% of patients).

Finally, they decide there will be no way to distinguish trial‐specific intercurrent events from those intercurrent events that would occur in routine clinical practice.

*Choice of estimand(s)*

Because trial‐specific intercurrent events are likely, but cannot be identified, investigators decide to use two estimands: (i) one which assumes there are no trial‐specific intercurrent events, where they use a treatment policy strategy to handle deviations from the assigned transfusion strategy; and (ii) one which uses a hypothetical strategy to handle deviations from the assigned transfusion strategy.

Investigators decide that even 5% of patients experiencing trial‐specific intercurrent events would be enough to increase the risk of a spurious non‐inferiority conclusion to an unacceptable level. Therefore, they decide to specify the two estimands (treatment policy and hypothetical) as co‐primary estimands, and require a demonstration of non‐inferiority for both.

*Choice of estimator*

Investigators consider using either IPW (which requires the inclusion of all confounders in the model) or *IV(Bayes)* (which requires correct specification of the prior for the effect of the liberal transfusion arm vs. transfusing at a higher platelet count, and that the treatment effect under hypothetical compliance be the same across compliance levels).

For the *IV(Bayes)* approach, investigators feel confident that there will be little difference between the liberal transfusion threshold and transfusing at a higher threshold, that is, they feel confident they can specify an accurate prior. Further, based on prior research and biological plausibility, they feel it is likely the treatment effect (restrictive vs. liberal threshold) under hypothetical compliance will be the same across all patients.

Though investigators feel that based on prior research they could identify and include all likely confounders for the IPW approach, they feel more confident in the assumptions for the *IV(Bayes)* approach. Therefore, they choose this as their estimator. They specify a number of sensitivity analyses under which they specify smaller or larger effects for the prior.

*Impact on sample size requirements*

Investigators decide to inflate their sample size to allow for the anticipated level of deviations from the assigned transfusion strategy, to ensure the study is well powered for the hypothetical strategy estimand. Because they will be using a hypothetical strategy for *all* deviations (both trial‐specific and non‐trial‐specific, as they cannot distinguish between the two), this is based on the total proportion of anticipated deviations (around 10%), not the anticipated proportion of trial‐specific intercurrent events (which, as above, is around 5%).

Therefore, investigators decide to use co‐primary estimands (one assuming no trial‐specific intercurrent events, using a treatment policy strategy, and one assuming all deviations from the assigned threshold are trial‐specific, using a hypothetical strategy), and require non‐inferiority to be shown for both. They increase their sample size to ensure they are well powered for the estimand using the hypothetical strategy (e.g., using simulation to obtain a plausible value of the standard error to use in the sample size calculation for the hypothetical estimator).

Finally, they decide to use *IV(Bayes)* to estimate the hypothetical strategy estimand, on the basis they are confident that (i) there will be little difference between the liberal transfusion threshold and transfusing at a higher threshold, so they can specify an accurate prior; and (ii) there is unlikely to be any treatment effect heterogeneity across compliance levels (i.e., the treatment effect if all patients were to comply is the same across all principal strata).

## 10 Discussion

Common advice for non‐inferiority trials is that ITT be supplemented by per‐protocol analyses as protection against the risk of erroneously declaring NI based on a proliferation of protocol deviations which makes treatment arms more similar than they would be in practice. However, there are two issues with this advice: (i) it is based on statistical considerations alone, and does not consider estimands; and (ii) per‐protocol analyses do not inherently protect against protocol deviations—as seen in TOPPS, they can actually increase the risk of erroneously declaring NI due to bias from post‐randomization exclusions. In this article we sought to address the above deficiencies by updating the advice in light of recent focus on estimands, and identifying and comparing methods of estimation which improve on per‐protocol.

We argue that non‐adherence or protocol deviations themselves are not inherently a threat to the validity of NI trials. Such intercurrent events occur in practice, and are thus simply something that needs to be defined as part of the estimand. Rather, the threat to validity comes from trial‐specific intercurrent events (those that occur in a trial setting but would not occur in practice, for instance due to poor study conduct). These intercurrent events can serve to make treatment arms more similar than they would be in practice, thus increasing the risk of declaring NI when the new intervention is in fact worse than control. For example, our simulation study found that ignoring trial‐specific intercurrent events when such events affected 10% of participants led to between two‐ and three‐fold increases in the type I error rate above its nominal level.

However, further complicating the issue is that intercurrent events that would occur in practice cannot always be distinguished from those that would not. In TOPPS, for example, some degree of non‐compliance to the transfusion policies would be expected in practice, albeit to a lesser degree than that seen in the trial, but there is no way to distinguish which category any particular deviation falls under.

We therefore suggest for NI trials where trial‐specific intercurrent events may be an issue, they be handled in the estimand using a hypothetical strategy. The hypothetical estimand serves as reassurance that a NI conclusion is real and not due to trial‐specific issues. The strategies for other, non‐trial‐specific, intercurrent events could be based on clinical considerations. We note that our advice does not prohibit the use of the hypothetical strategy for non‐trial‐specific intercurrent events if clinically warranted. We also note that the underlying factors that introduced trial‐specific intercurrent events may also affect other aspects of the trial, and warrants careful consideration by investigators.

Estimation of hypothetical treatment effects can be challenging and requires untestable assumptions. Using simulation and a re‐analysis of TOPPS, we evaluated several methods that could be used for NI trials with non‐compliance in both treatment arms. We found that IPW and *IV(Bayes)* are good options provided their underlying assumptions are fulfilled. Conversely, *IV(interaction)* did not perform well in the scenarios considered in our simulation study, and so we cannot see any advantage to using it over either IPW or *IV(Bayes)*. A key assumption for the *IV(interaction)* method is that there is an interaction between treatment arm and the covariate in terms of compliance, and the strength of this interaction determines the precision of the estimates. This condition was met in compliance scenarios 2b, 2c, and 3b, with large interactions of 20% point difference in compliance probabilities. However, it seems that this size of interaction is not sufficient for this method to perform well. The per‐protocol analysis also performed well in certain scenarios, though it overestimated the treatment effect and did not maintain the type I error rate when there was a large degree of treatment effect heterogeneity and large differences in compliance between treatment arms. The per‐protocol analysis also performed poorly in the re‐analysis of TOPPS. As the assumptions behind the per‐protocol analysis are highly likely to be violated in trials of time‐varying treatments, we do not recommend its use.

The choice between IPW and *IV(Bayes)* could be made based on which set of assumptions are more plausible for a given trial. For many NI trials information on the standard treatment is available from previous studies, which could inform choice of prior for *IV(Bayes)*. However, careful consideration is required as to whether effects from previous studies will apply to the current study—if not, this could induce bias. For IPW, consideration needs to be given to potential confounders between compliance status and outcomes, and such potential confounders need to be collected during the study to be used during estimation.

Recently, Lynggaard et al. also considered the choice of estimand in NI trials [39]. They argue that co‐primary estimands should not be applied in NI trials simply to reflect the historical approach of using ITT and PP analyses, and argue that in most cases, NI trials should have a single primary estimand based on clinical objectives, as would be the case for most superiority trials. However, they acknowledge that multiple estimands may be appropriate for certain trials. In this article, we expand on this view to provide guidance on when multiple estimands are necessary and the factors that it might depend on, with these arguments being based primarily on the occurrence of trial‐specific IEs (see Tables 1 and 2).

In this work, we have argued that non‐trial‐specific intercurrent events do not pose a specific threat to the validity of NI trials, as they do not make treatment arms artificially more similar. However, such intercurrent events can still pose issues around the interpretation of results, as in any other trial. For instance, if a new treatment is only non‐inferior on the basis that most patients switch to the more effective standard treatment during the trial, its use in routine care may not be warranted (even if such switching would occur in practice). Thus, in this setting it may be useful to use a hypothetical strategy for treatment switching when defining the estimand, or, alternatively, using a smaller non‐inferiority margin to account for the anticipated degree of switching.

In this work we have focussed on estimation of a hypothetical strategy to guard against spurious conclusions of non‐inferiority driven by trial‐specific intercurrent events. However, spurious conclusions of non‐inferiority can also be driven by other factors, such as missing outcome data, even in the absence of trial‐specific events. For instance, even when a treatment policy strategy is used, some methods of handling missing data may be biased or lead to incorrect standard errors, and therefore increase the risk of a spurious conclusion. Evaluation of methods to estimate a treatment policy strategy in the presence of missing data is an active research area [40, 41, 42, 43]; however, it would be useful for future research efforts to focus on evaluating these methods specifically in the non‐inferiority setting.

There are some limitations to this work. First, our simulation study only considered the setting with a continuous outcome and all‐or‐nothing compliance, and thus our results may not be generalisable to other outcome or compliance types. Second, although we generated plausible interactions in our simulation study, they may not have been sufficiently large for the *IV(interaction)* approach. Thus, our results may not apply to settings with larger interactions. Third, while we considered a wide range of parameter values in our simulation study, it is hard to assess how realistic some of these choices are since many of these relate to unmeasurable quantities (e.g., the association between the outcome and the unmeasured confounder). Fourth, we only considered a frequentist evaluation of the *IV(Bayes)* method. Fifth, for IPW we did not consider methods to account for uncertainty in estimating the weights when calculating standard errors. Although we found that Stata's default sandwich estimator performed well in simulation studies, this may not be the case in other settings, and so evaluation of methods to account for such uncertainty, such as the non‐parametric bootstrap, would be useful. Finally, we only considered the setting where a single binary covariate was included in IPW models. Inclusion of more variables may affect performance. For instance, IPW models dropped some observations due to perfect predictions in settings with high compliance; this issue may be exacerbated when more variables are included in the model.

The results here suggest a number of areas for future work. We have focussed primarily on defining estimands and estimators for non‐inferiority trials, though it would be useful to evaluate how the estimands framework should impact on choice of non‐inferiority margin. Further, as mentioned above, our simulation study focussed only on a continuous outcome with all‐or‐nothing compliance. It would be useful to evaluate these estimators in a wider range of settings (e.g., for binary or time‐to‐event outcomes, for different types of intercurrent events such as treatment switching or use of rescue medication, and for treatments which are time‐varying, such as in TOPPS, rather than all‐or‐nothing). Further, our focus was primarily on the bias of different estimators. It may be useful to compare different approaches to calculating standard errors for each approach. IPW has been well studied in many of these settings [25], so may be a preferable option in such contexts until *IV(Bayes)* has been more fully evaluated. Evaluation of some additional methods may be useful. For instance, following on from the results from Lasch et al., who found that *g‐estimation* can retain nominal power in some settings [44, 45], a comparison between this and IPW and *IV(Bayes)* would be useful. Further, evaluation of methods which make similar assumptions to IPW (such as multiple imputation and doubly‐robust methods [23]) may be useful to determine whether there is any benefit to using these alternative approaches. Finally, this work may have implications for reporting guidelines. For instance, future updates of the CONSORT guidance for non‐inferiority trials should consider including reporting items such as how trial‐specific intercurrent events are handled in the choice of estimand, how they are handled in the estimator, and whether any observed intercurrent events that occur during the trial were suspected to be trial‐specific.

To conclude, in non‐inferiority trials, trial‐specific intercurrent events can make treatment arms more similar than they would be in practice, thus increasing the risk of erroneously declaring NI. To guard against this, an estimand using a hypothetical strategy for trial‐specific intercurrent events should be used. IPW and *IV(Bayes)* may both be good options for estimating hypothetical effects when there is all‐or‐nothing compliance in two treatment arms.

## Author Contributions

K.E.M., I.R.W., C.L., and B.C.K. designed the simulation study. K.E.M. implemented the simulation study. B.C.K. re‐analyzed the TOPPS data. K.E.M. and B.C.K. wrote the first draft of the manuscript. I.R.W., C.L., and S.S. revised the manuscript. All authors read and approved the final manuscript.

## Conflicts of Interest

The authors declare no conflicts of interest.

## Supporting information


**Data S1.** Supporting Information.

**Appendix S1.** Supporting Information.

## Data Availability

Data sharing requests for the TOPPS data must be directed towards the trial sponsor.
