# Supplementary material for: Applying the Estimands Framework to Non‐Inferiority Trials: Guidance on Choice of Hypothetical Estimands for Non‐Adherence and Comparison of Estimation Methods
Source: Stat Med. 2025 Feb 7;44(5):e10348. doi: 10.1002/sim.10348 (PMC11806244; doi:10.1002/sim.10348)
Supplement: Supplementary file 2 — Appendix S1. Supporting Information. [file SIM-44-0-s002.docx]

**Supplementary appendix:** **Applying the estimands framework to non-inferiority trials: guidance on choice of hypothetical estimands for non-adherence and comparison of estimation methods**

**Contents**

|  | Page |
| --- | --- |
| 1. Results from TOPPS trial | 1 |
| Example Stata code to implement estimators for hypothetical estimand | 2 |
| 1. Details of simulation study | 3 |
| 1. Description of methods for re-analysis of TOPPS | 8 |
| 1. Tables for simulations results (all scenarios) | 10 |

# Results from TOPPS trial

**Table S1 – Analysis results for the primary outcome (bleeding) from the TOPPS trial**

| **Analysis method** | **Non-prophylactic group**  **(new treatment) – no. (%)** | **Prophylactic group**  **(standard treatment) – no. (%)** | **Difference in percentage points^a^ (90% CI)** | **Non-inferiority demonstrated** |
| --- | --- | --- | --- | --- |
| ITT | 151/300 (50) | 128/298 (43) | 8.4 (1.7 to 15.2) | No |
| PP | 123/258 (48) | 101/230 (44) | 4.5 (-3.0 to 12.0) | Yes |

^a^ Treatment effect estimate was adjusted for diagnosis and treatment plan as minimisation factors

**Table S2 – Primary outcome events by treatment group for those included vs. excluded from the per-protocol analysis in the TOPPS trial**

|  | **Non-prophylactic group**  **(new treatment)** | **Prophylactic group**  **(standard treatment)** |
| --- | --- | --- |
| Included in per-protocol analysis (n=488) | 123/258 (48) | 101/230 (44) |
| Excluded from per-protocol analysis (n=110) | 28/42 (67) | 27/68 (40) |

# Example Stata code to implement estimators for hypothetical estimand

**Table S3 – Example Stata code used to implement estimators in trials with a continuous outcome and ‘all-or-nothing’ compliance in both treatment arms**

| **Estimator** | **Stata v14 code used to implement estimator^a^** |
| --- | --- |
| ITT | reg y z x |
| Per protocol | reg y z x if c == 1 |
| IPW | gen dev_trt_z0 = 1 – c_z0 if z == 0  gen dev_trt_z1 = 1 – c_z1 if z == 1  logistic dev_trt_z0 x if z == 0  predict prob_dev_trt_z0 if z == 0  logistic dev_trt_z1 if z == 1  predict prob_dev_trt_z1 if z == 1  gen prob_dev = prob_dev_trt_z0 if z == 0  replace prob_dev = prob_dev_trt_z1 if z == 1  gen weight = 1/(1-prob_dev)  reg y z [pw=weight] if c == 1 |
| IV(Bayes) | reg c_z1 z x  predict prob_new_trt  reg c_z0 z x  predict prob_std_trt  bayesmh y prob_new_trt prob_std_trt x, likelihood(normal({var})) ///  prior({y: _cons prob_new_trt x}, flat) ///  prior({y: prob_std_trt}, normal(*mean*, *sd*)) ///  prior({var}, jeffreys)  bayesstats summary ({y:prob_new_trt}-{y:prob_std_trt}) |
| IV(interaction) | ivregress 2sls y x (c_z1 c_z0= z z#x)  lincom c_z1 - c_z0 |

^a^ Where: y=patient outcome, z=treatment allocation, x=observed covariate, c=compliance status (1=yes, 0=no), c_z0=complied to treatment z=0 (and equal to 0 for patients allocated to treatment 1), c_z1=complied to treatment z=1 (and equal to 0 for patients allocated to treatment 0)

# Details of simulation study

We performed two simulations studies: the first when there is no treatment effect heterogeneity across compliance levels, and the second which did include treatment effect heterogeneity across compliance levels.

All simulation scenarios were based on a two-arm randomised non-inferiority trial with a continuous outcome. Non-compliance occurred in both treatment arms, and compliance was ‘all or nothing’, i.e. patients either received their allocated treatment or received nothing.

We conducted the simulations using Stata, and used 5000 replications for each scenario. Stata code used to simulate data is available in the supplementary material for two scenarios (one each for simulation studies 1 and 2), and code for the other scenarios was identical except for modifications to the input parameters.

Below we summarise the key aspects of the two simulation studies.

### Simulation study 1 (no treatment effect heterogeneity)

#### Data generating models

We generated data using two models. First, we generated each patient’s probability of complying with their allocated treatment on a linear scale using the following model (regression parameters represent differences in proportions):

$$P\left( C=1 \right)= \alpha^{C}+\beta_{Z}^{C}Z+\beta_{X}^{C}X+\beta_{U}^{C}U+\beta_{ZX}^{C}ZX+\beta_{ZU}^{C}ZU (8)$$

We generated compliance probability on a linear scale (rather than logistic) to match models 2-3 and 5-6 (main paper). The terms $X$ and $U$ are used in this model to produce confounding between compliance status and outcome (where $X$ is an observed baseline covariate, and so produces measured confounding, and $U$ is an unobserved baseline covariate, and so produces unmeasured confounding). Observed compliance status, $C$, was then generated from a Bernoulli distribution with probability from model 8.

We generated $Z$ by randomly allocating half of the patients into each treatment group. Both $X$ and $U$ were drawn independently from a Bernoulli distribution with probability 0.5.

Second, we generated each patient’s outcome based on the following model:

$$Y= \alpha^{Y}+\beta_{C0}^{Y}C\left( 1-Z \right)+\beta_{C1}^{Y}CZ+\beta_{X}^{Y}X+\beta_{U}^{Y}U+\varepsilon(9)$$

where $\varepsilon$ is normally distributed with mean 0 and variance $\sigma_{\epsilon}^{2}$. The second and third terms in this model represent causal effects of receiving treatments 0 and 1 respectively. Note that lower values of $Y$ imply poorer outcomes.

#### Estimand

The hypothetical estimand, $E\left( Y^{\left( Z=1, C=1 \right)} \right)-E\left( Y^{\left( Z=0, C=1 \right)} \right)$, is equivalent to the quantity $\beta_{C1}^{Y}-\beta_{C0}^{Y}$ from model (9).

#### Simulation scenarios

We considered five scenarios (labelled A-E, shown in Table S4) in which we varied the sample size, percentage compliance, true treatment effect, and association between covariates $X$ and $U$ and outcome. Values of the estimand for each scenario are provided in Table S4. Then, for each of these five scenarios, we also considered eight compliance scenarios (labelled 1, 2a-c, 3a-b, and 4a-b; shown in Table 3 and Table S5). This led to a total of 5x8=40 scenarios. We used a non-inferiority margin of -0.3 in all scenarios.

The aims of scenarios A-E were to assess the impact of smaller vs. larger sample sizes, smaller vs. larger degrees of non-compliance, smaller vs. larger associations between covariates and the outcome, as well as the impact on power. We set the true treatment effect ($\beta_{C1}^{Y}-\beta_{C0}^{Y}$) to -0.3 in scenarios A-D in order to evaluate the type I error rate (i.e. -0.3 was the null hypothesis in these scenarios), and to 0 in scenario E in order to evaluate power.

The aims of compliance scenarios 1-4 were to evaluate the impact of different types of compliance. We evaluated scenarios where compliance was completely random, and did not depend on any measured or unmeasured baseline covariates (scenario 1); compliance depended only on measured baseline covariates (scenario 2); compliance depended both on measured and unmeasured baseline covariates (scenario 3); and compliance depended only on unmeasured baseline covariates (scenario 4). For scenarios 2-4, we also evaluated scenarios where the compliance-outcome association was either confounded or unconfounded by baseline covariates. A description of the different compliance scenarios is given in Table 3, and the parameter values used for each compliance scenario is given in Table S5.

We fixed the following parameter values for all scenarios: $\alpha^{Y}=0$, $\beta_{C0}^{Y}=0.6$ (implying the effect of the standard treatment vs no treatment is 0.6), and $\sigma_{\epsilon}^{2}=1$.

**Analysis models**

We implemented five estimators; (i) intention-to-treat; (ii) per-protocol; (iii) IPW; (iv) *IV(Bayes)*; and (v) *IV(interaction)*. All analyses adjusted for the observed covariate $X$ (except for IPW, which used $X$ to estimate weights).

For *IV(Bayes)*, we evaluated four different normally distributed priors for the effect of standard treatment vs no treatment, $\beta_{C0}^{Y}$ (where the true value of this effect is 0.6):

1. Well centred, precise prior (mean 0.6, standard deviation (SD) 0.2)
2. Well centred, vague prior (mean 0.6, SD 2.0)
3. Miscentred, precise prior (mean 1.2, SD 0.2)
4. Miscentred, value, vague prior (mean 1.2, SD 2.0)

We used these four priors to evaluate the impact of misspecifying the prior for $\beta_{C0}^{Y}$, as well as the impact of more vs less precise priors. For our precise prior, we set the standard deviation to be equivalent to the standard error for the treatment effect estimate from a previous trial comparing the standard treatment to placebo/no treatment, with a total sample size of 100, with residual standard deviation of 1. For the imprecise prior, we set the standard deviation to be 10 times the SD from the precise prior.

In some scenarios, estimates and standard errors for the *IV(interaction)* approach were very unstable, leading to extreme outliers which greatly affected summaries. This was due to near-collinearity in the second stage of the two-stage least squares procedure. Because this can sometimes be detected by tests for under-identification we therefore report four summaries of the *IV(interaction)* results: (a) including all 5000 replications; (b) only including replications where the SE from the *IV(interaction)* approach was less than 100 times the SE from the ITT approach, i.e. SE(*IV(interaction)*)<100xSE(ITT); (c) only including replications where SE(*IV(interaction)*)<10xSE(ITT); and (d) only including replications where p<0.05 for a test of underidentification. The aim of reporting these additional summaries was to evaluate the *IV(interaction)* approach when it had ‘converged’ to a sensible solution.

**Performance measures**

For each analysis method, we assessed bias in the estimated treatment effect, the percentage difference between the model-based and empirical SEs (as a measure of bias of the estimated SE), and the ratio of the empirical SE for each method relative to the empirical SE for the intention-to-treat method (to evaluate precision). We calculated the type I error rate for scenarios A-D as the proportion of replications in which non-inferiority was declared (i.e. the lower limit of the 95% confidence interval was above the non-inferiority margin). We calculated power for scenario E as the proportion of replications in which non-inferiority was declared. The nominal type I error rate was set to 2.5%, and so rates above this level are considered inflated.

### Simulation study 2 (treatment effect heterogeneity)

We used two treatment effect heterogeneity (TEH) scenarios: one in which the treatment effect varied across values of $X$ (which implies it varies across compliance status, as $X$ is strongly associated with compliance), and one in which it varied across values of $U$ (which also implies it varies across compliance status; the key difference between these scenarios is that $X$ is observed while $U$ is not). We label these two scenarios *TEH(X) and TEH(U)*.

Both TEH scenarios used model (8) for generating compliance but generated outcome data differently (in order to include the treatment effect heterogeneity in the generated outcomes).

*TEH(X)* used compliance scenario 2c for the moderate difference in compliance scenario (see below for parameters for the large difference in compliance scenario), and the data generating mechanism for the outcome $Y$ was:

$$Y= \alpha^{Y}+\beta_{C0}^{Y}C\left( 1-Z \right)+\beta_{C1,X=0}^{Y}\left( 1-X \right)CZ+\beta_{C1,X=1}^{Y}XCZ+\beta_{X}^{Y}X+\beta_{U}^{Y}U+\varepsilon$$

where $\beta_{C1,X=0}^{Y}$ and $\beta_{C1,X=1}^{Y}$ denote the effect of receiving the new treatment ($Z=1$ and C=1) against no treatment for $X=0$ and $1$ respectively (implying the treatment effect is $\beta_{C1,X=0}^{Y}-\beta_{C0}^{Y}$ for $X=0$, and $\beta_{C1,X=1}^{Y}-\beta_{C0}^{Y}$ for $X=1$, with the value of the hypothetical estimand then being an average of the values across $X$). We used the same values of $\alpha^{Y}$, $\beta_{X}^{Y}$, $\beta_{U}^{Y}$, and $\beta_{C0}^{Y}$ as for scenario B in simulation study 1.

We varied values for two factors for the *TEH(X)* scenario: the degree of TEH (moderate vs. large), and the difference in compliance between treatment groups (moderate vs. large). Moderate differences in compliance were defined as $P\left( C=1 \right)=$0.65 for $Z=0$ and 0.75 for $Z=1$ (i.e. using the parameter values for compliance scenario 2c: $\alpha^{C}=0.7, \beta_{X}^{C}=-0.1,\beta_{ZX}^{C}=0.2$), and large differences were defined as $P\left( C=1 \right)=$0.6 for $Z=0$ and 0.8 for $Z=1$ (achieved by changing the parameters in the compliance generation model to: $\alpha^{C}=0.7, \beta_{X}^{C}=-0.2, \beta_{ZX}^{C}=0.4$). Moderate differences in degree of TEH were defined as values of $\beta_{C1,X=0}^{Y}-\beta_{C0}^{Y}=-0.15$ and $\beta_{C1,X=1}^{Y}-\beta_{C0}^{Y}=-0.45$, and large differences were defined as values of 0 for $X=0$ and -0.6 for $X=1$. Because $X$ occurs with probability 0.5, this implies the hypothetical estimand value for *TEH(X)* is -0.3 for both moderate and large degrees of TEH (the same as in simulation study 1).

*TEH(U)* was based on a modified version of compliance scenario 2c to allow differential compliance across values of $U$ instead of $X$; we set $\alpha^{C}=0.7$; $\beta_{U}^{C}=-0.1$, $\beta_{ZU}^{C}=0.2$ (for moderate differences in compliance) or $\beta_{U}^{C}=-0.2, \beta_{ZU}^{C}=0.4$ (for large differences in compliance); and $\beta_{X}^{C}=\beta_{ZX}^{C}=0$ (i.e. $U$ acted as an unmeasured confounder between compliance and outcome, and compliance varied across values of $U$ with healthier patients being less likely to comply with the standard treatment and more likely to comply with the new treatment, and compliance being higher in the new treatment group). The data generating mechanism for the outcome $Y$ was:

$$Y= \alpha^{Y}+\beta_{C0}^{Y}C\left( 1-Z \right)+\beta_{C1,U=0}^{Y}\left( 1-U \right)CZ+\beta_{C1,U=1}^{Y}UCZ+\beta_{X}^{Y}X+\beta_{U}^{Y}U+\varepsilon$$

We varied factors in the same way as for *TEH(X)* (i.e. moderate vs. large degrees of TEH, and moderate vs. large differences in compliance. We used the same parameter values for these scenarios as for *TEH(U)*, except using $\beta_{C1,U=0}^{Y}$ and $\beta_{C1,U=1}^{Y}$ instead of $\beta_{C1,X=0}^{Y}$ and $\beta_{C1,X=1}^{Y}$.

**Table S4 - Summary of scenarios A-E and parameter values used**

| **Scenario** | **Number of patients (total)** | **Overall percentage compliance** | **Value of** $\boldsymbol{\beta}_{\boldsymbol{X}}^{\boldsymbol{Y}}$ **and** $\boldsymbol{\beta}_{\boldsymbol{U}}^{\boldsymbol{Y}}$**^a^** | **Value of estimand (**$\boldsymbol{\beta}_{\mathbf{C1}}^{\boldsymbol{Y}}\mathbf{-}\boldsymbol{\beta}_{\mathbf{C0}}^{\boldsymbol{Y}}$**)** |
| --- | --- | --- | --- | --- |
| A | 100 | 70 | 1 | -0.3 |
| B | 500 | 70 | 1 | -0.3 |
| C | 500 | 90 | 1 | -0.3 |
| D | 100 | 70 | 0.2 | -0.3 |
| E | 100 | 70 | 1 | 0 |

^a^ We set $\beta_{X}^{Y}=\beta_{U}^{Y}$ for all scenarios

**Table S5 – Parameter values used for compliance scenarios for simulation study 1 (values represent differences in proportions)**

| **Compliance scenario** | **Overall percentage compliance** | $\boldsymbol{\alpha}^{\boldsymbol{C}}$ | $\boldsymbol{\beta}_{\boldsymbol{Z}}^{\boldsymbol{C}}$ | $\boldsymbol{\beta}_{\boldsymbol{X}}^{\boldsymbol{C}}$ | $\boldsymbol{\beta}_{\boldsymbol{U}}^{\boldsymbol{C}}$ | $\boldsymbol{\beta}_{\boldsymbol{ZX}}^{\boldsymbol{C}}$ | $\boldsymbol{\beta}_{\boldsymbol{ZW}}^{\boldsymbol{C}}$ |
| --- | --- | --- | --- | --- | --- | --- | --- |
| 1 | 70 | 0.7 | 0 | 0 | 0 | 0 | 0 |
|  | 90 | 0.9 | 0 | 0 | 0 | 0 | 0 |
| 2a | 70 | 0.7 | 0.1 | -0.1 | 0 | 0 | 0 |
|  | 90 | 0.9 | 0.05 | -0.05 | 0 | 0 | 0 |
| 2b | 70 | 0.75 | -0.1 | -0.1 | 0 | 0.2 | 0 |
|  | 90 | 0.95 | -0.1 | -0.1 | 0 | 0.2 | 0 |
| 2c | 70 | 0.7 | 0 | -0.1 | 0 | 0.2 | 0 |
|  | 90 | 0.925 | -0.05 | -0.1 | 0 | 0.2 | 0 |
| 3a | 70 | 0.75 | 0.1 | -0.1 | -0.1 | 0 | 0 |
|  | 90 | 0.9 | 0.05 | -0.025 | -0.025 | 0 | 0 |
| 3b | 70 | 0.80 | -0.2 | -0.1 | -0.1 | 0.2 | 0.2 |
|  | 90 | 0.95 | -0.1 | -0.05 | -0.05 | 0.1 | 0.1 |
| 4a | 70 | 0.70 | 0.1 | 0 | -0.1 | 0 | 0 |
|  | 90 | 0.90 | 0.05 | 0 | -0.05 | 0 | 0 |
| 4b | 70 | 0.75 | -0.1 | 0 | -0.1 | 0 | 0.2 |
|  | 90 | 0.95 | -0.1 | 0 | -0.1 | 0 | 0.2 |

# Description of methods for re-analysis of TOPPS

We adjusted for several baseline covariates (relapsed disease, previous stem cell transplantation, fungal infection, and organ failure) in both the intention-to-treat and per-protocol analysis. For the per-protocol analysis, we excluded any patients with a deviation.

For IPW, we analysed each study day separately (to allow for the fact that non-compliance could occur at any point during the study period). We excluded data after the first deviation, then used inverse probability weighting to account for potential confounding between deviations and outcome. Weights were calculated using two post-randomisation variables (whether the patient had previously experienced a minor bleed during follow-up [WHO grade 1], and whether the patient had previously experienced a major bleed during follow-up [WHO grade 2-4]). The model further adjusted for several baseline covariates (relapsed disease, previous stem cell transplantation, fungal infection, and organ failure). We used standardised weights with robust standard errors. Because the model included each study day (prior to deviation) as separate observations, we estimated the average effect across all study days, then transformed the estimate to match the original outcome (by multiplying the coefficient by 30).

For *IV(interaction)* we fit four separate models, each using a different baseline covariate as an instrument. This was to evaluate the impact that different choices in baseline covariate had on results. The four baseline covariates were: (i) relapsed disease; (ii) previous stem cell transplantation (SCT); (iii) fungal infection; and (iv) organ failure. The interaction between each covariate and treatment allocation on compliance is shown in table S6.

For the *IV(Bayes)* approach we fit four separate models, each using a different prior for the effect of the prophylactic strategy (the active control). This was to evaluate the impact that different choices of prior had on results. Here, the prior does not represent the effect of the prophylactic strategy vs. no treatment (as was the case in the simulation study), but instead represents the effect of the prophylactic strategy vs. deviating from the prophylactic strategy, which implies being transfused at a higher threshold than the strategy required.

We chose these priors based on our judgement of what was plausible; this was done specifically for the purpose of this re-analysis, and so they were chosen retrospectively after the trial was already complete. For the four normally distributed priors, we used combinations of small vs large effects and precise vs vague variances. We chose a large effect as an increase of 2 days with bleeding, on the basis that following the prophylaxis strategy leads to lower platelet counts than deviating from it (i.e. transfusing before the platelet count dropped below the prophylaxis threshold), and lower platelet counts may increase the risk of bleeding. We chose a small effect as no difference in days with bleeding, under the assumption that so long as the platelet count is above the prophylaxis threshold, there is little difference in the risk of bleeding. We used precise and vague variances of 1 and 10 respectively.

**Table S6 – Interaction between baseline characteristics and treatment arm on compliance in TOPPS (for *IV(interaction)* method)**

| **Baseline characteristic** | **Estimate of interaction between baseline covariate and treatment group* (95% CI)** |
| --- | --- |
| Relapsed disease | 3.2 (-10.0, 16.3) |
| Previous SCT | 1.7 (-22.3, 25.6) |
| Fungal infection | -9.0 (-57.9, 40.0) |
| Organ failure | 18.5 (-5.8, 42.8) |

*Estimated from a generalised linear model with an identity link and Binomial family, with compliance as the outcome and treatment, baseline covariate, and their interaction as independent variables. Interaction terms represent a difference in percentage points

# Tables for simulations results (all scenarios)

**Scenario A; compliance scenario 1**

| Analysis method | Mean of estimates | Empirical SE | Model-based SE | % bias in model-based SE | Type I error^1^ | Percentage increase in precision (ITT vs alternative)^2^ |
| --- | --- | --- | --- | --- | --- | --- |
| Intention to treat^3^ | -0.211 | 0.229 | 0.229 | -0.0 | 5.9 |  |
| Per protocol | -0.298 | 0.270 | 0.271 | 0.3 | 2.4 | 39.0 |
| IPW (all) | -0.299 | 0.291 | 0.296 | 1.6 | 2.4 | 61.5 |
| IPW (4992)^4^ | -0.299 | 0.290 | 0.296 | 2.0 | 2.4 | 60.4 |
| IV(Bayes) centred precise prior | -0.302 | 0.326 | 0.340 | 4.5 | 2.3 | 102.5 |
| IV(Bayes) centred vague prior | -0.302 | 0.326 | 0.387 | 18.7 | 1.4 | 102.6 |
| IV(Bayes) miscentred precise prior | -0.297 | 0.336 | 0.340 | 1.3 | 2.6 | 115.6 |
| IV(Bayes) miscentred vague prior | -0.297 | 0.336 | 0.386 | 15.1 | 1.7 | 114.9 |
| IV(interaction) (all) | -0.218 | 10.839 | 4745.008 | 43675.2 | 0.4 | 224009.6 |
| SE <= 100 SE(ITT) (4684)^5^ | -0.301 | 0.742 | 3.468 | 367.4 | 0.4 | 950.0 |
| SE <= 10 SE(ITT) (3908)^6^ | -0.306 | 0.444 | 0.852 | 92.0 | 0.5 | 275.8 |
| p < 0.05 (253)^7^ | -0.290 | 0.349 | 0.377 | 7.9 | 1.6 | 132.2 |

^1^Type I error calculated using a t-distribution with n-3 degrees of freedom for ITT and PP (where n is the number of patients included in the regression model for PP), n-2 degrees of freedom for IPW, n-4 degrees of freedom for IV, and using the 95% credible interval for the Bayes methods; ^2^$100\left( \left( \frac{Empirical SE\left( \mathrm{alternative} \right)}{Empirical SE\left( \mathrm{ITT} \right)} \right)^{2}-1 \right)$; ^3^Note that the Intention to treat estimator is targeting a different estimand to the other estimators (treatment policy), with a different true value of the estimand; ^4^Number of IPW regressions with no observations dropped due to perfect prediction in the logistic regression generating weights; ^5^IV(interaction): regression summarized for those runs with a SE less than or equal to 100 times the ITT empirical SE. Number of runs included given in brackets; ^6^IV regression summarized for those runs with a SE less than or equal to 10 times the ITT empirical SE; ^7^IV regression summarized for those runs with p<0.05 from the test for under-identification

**Scenario A; compliance scenario 2a**

| Analysis method | Mean of estimates | Empirical SE | Model-based SE | % bias in model-based SE | Type I error | Percentage increase in precision (ITT vs alternative) |
| --- | --- | --- | --- | --- | --- | --- |
| Intention to treat | -0.167 | 0.221 | 0.229 | 3.8 | 7.3 |  |
| Per protocol | -0.304 | 0.266 | 0.272 | 2.2 | 2.3 | 44.9 |
| IPW (all) | -0.300 | 0.289 | 0.298 | 3.2 | 2.3 | 70.8 |
| IPW (4963) | -0.303 | 0.286 | 0.298 | 4.2 | 2.1 | 67.2 |
| IV(Bayes) centred precise prior | -0.303 | 0.293 | 0.321 | 9.5 | 1.7 | 76.4 |
| IV(Bayes) centred vague prior | -0.303 | 0.294 | 0.397 | 35.1 | 1.1 | 77.0 |
| IV(Bayes) miscentred precise prior | -0.379 | 0.303 | 0.321 | 6.2 | 1.3 | 87.8 |
| IV(Bayes) miscentred vague prior | -0.379 | 0.303 | 0.397 | 31.1 | 0.9 | 87.8 |
| IV(interaction) (all) | -1.205 | 26.783 | 7342.502 | 27315.2 | 0.3 | 1471090.9 |
| SE <= 100 SE(ITT) (4607) | -0.306 | 0.934 | 3.850 | 312.3 | 0.4 | 1687.6 |
| SE <= 10 SE(ITT) (3619) | -0.298 | 0.491 | 0.905 | 84.1 | 0.5 | 395.3 |
| p < 0.05 (286) | -0.287 | 0.375 | 0.410 | 9.1 | 0.7 | 188.9 |

**Scenario A; compliance scenario 2b**

| Analysis method | Mean of estimates | Empirical SE | Model-based SE | % bias in model-based SE | Type I error | Percentage increase in precision (ITT vs alternative) |
| --- | --- | --- | --- | --- | --- | --- |
| Intention to treat | -0.214 | 0.230 | 0.229 | -0.5 | 5.6 |  |
| Per protocol | -0.304 | 0.270 | 0.271 | 0.4 | 2.6 | 37.7 |
| IPW (all) | -0.305 | 0.289 | 0.296 | 2.6 | 2.4 | 57.6 |
| IPW (4987) | -0.304 | 0.288 | 0.296 | 3.0 | 2.4 | 56.3 |
| IV(Bayes) centred precise prior | -0.305 | 0.325 | 0.340 | 4.8 | 2.0 | 99.1 |
| IV(Bayes) centred vague prior | -0.305 | 0.325 | 0.387 | 19.0 | 1.4 | 99.7 |
| IV(Bayes) miscentred precise prior | -0.299 | 0.333 | 0.340 | 2.1 | 2.0 | 109.6 |
| IV(Bayes) miscentred vague prior | -0.299 | 0.333 | 0.387 | 16.0 | 1.5 | 110.0 |
| IV(interaction) (all) | 0.109 | 20.046 | 7540.578 | 37516.3 | 0.6 | 758793.1 |
| SE <= 100 SE(ITT) (4827) | -0.299 | 0.625 | 2.782 | 345.1 | 0.6 | 637.5 |
| SE <= 10 SE(ITT) (4339) | -0.305 | 0.418 | 0.703 | 67.9 | 0.7 | 230.8 |
| p < 0.05 (920) | -0.299 | 0.338 | 0.370 | 9.5 | 1.6 | 115.7 |

**Scenario A; compliance scenario 2c**

| Analysis method | Mean of estimates | Empirical SE | Model-based SE | % bias in model-based SE | Type I error | Percentage increase in precision (ITT vs alternative) |
| --- | --- | --- | --- | --- | --- | --- |
| Intention to treat | -0.166 | 0.227 | 0.229 | 1.0 | 7.9 |  |
| Per protocol | -0.300 | 0.271 | 0.272 | 0.5 | 2.4 | 42.3 |
| IPW (all) | -0.304 | 0.292 | 0.298 | 2.1 | 2.3 | 65.1 |
| IPW (4967) | -0.300 | 0.288 | 0.298 | 3.5 | 2.3 | 60.4 |
| IV(Bayes) centred precise prior | -0.300 | 0.302 | 0.321 | 6.5 | 1.8 | 76.6 |
| IV(Bayes) centred vague prior | -0.301 | 0.302 | 0.397 | 31.5 | 1.1 | 76.6 |
| IV(Bayes) miscentred precise prior | -0.377 | 0.312 | 0.322 | 3.1 | 1.2 | 88.8 |
| IV(Bayes) miscentred vague prior | -0.376 | 0.313 | 0.397 | 27.1 | 0.9 | 89.3 |
| IV(interaction) (all) | -0.274 | 4.756 | 577.508 | 12043.3 | 0.6 | 43727.5 |
| SE <= 100 SE(ITT) (4772) | -0.308 | 0.781 | 3.126 | 300.4 | 0.6 | 1080.9 |
| SE <= 10 SE(ITT) (4153) | -0.306 | 0.474 | 0.763 | 60.9 | 0.7 | 335.9 |
| p < 0.05 (953) | -0.271 | 0.378 | 0.404 | 6.8 | 2.1 | 177.6 |

**Scenario A; compliance scenario 3a**

| Analysis method | Mean of estimates | Empirical SE | Model-based SE | % bias in model-based SE | Type I error | Percentage increase in precision (ITT vs alternative) |
| --- | --- | --- | --- | --- | --- | --- |
| Intention to treat | -0.165 | 0.229 | 0.227 | -0.9 | 8.4 |  |
| Per protocol | -0.298 | 0.268 | 0.271 | 1.0 | 2.2 | 37.5 |
| IPW (all) | -0.296 | 0.290 | 0.297 | 2.5 | 2.4 | 60.3 |
| IPW (4977) | -0.298 | 0.288 | 0.297 | 3.2 | 2.3 | 57.8 |
| IV(Bayes) centred precise prior | -0.299 | 0.307 | 0.319 | 3.7 | 2.5 | 80.1 |
| IV(Bayes) centred vague prior | -0.299 | 0.307 | 0.394 | 28.6 | 1.5 | 79.5 |
| IV(Bayes) miscentred precise prior | -0.375 | 0.319 | 0.318 | -0.2 | 1.7 | 94.2 |
| IV(Bayes) miscentred vague prior | -0.375 | 0.319 | 0.394 | 23.6 | 1.3 | 94.1 |
| IV(interaction) (all) | 0.311 | 19.971 | 25730.312 | 128736.6 | 0.3 | 760735.4 |
| SE <= 100 SE(ITT) (4598) | -0.297 | 0.937 | 3.798 | 305.3 | 0.4 | 1575.2 |
| SE <= 10 SE(ITT) (3635) | -0.283 | 0.505 | 0.928 | 83.7 | 0.5 | 387.4 |
| p < 0.05 (259) | -0.298 | 0.363 | 0.411 | 13.2 | 0.4 | 151.7 |

**Scenario A; compliance scenario 3b**

| Analysis method | Mean of estimates | Empirical SE | Model-based SE | % bias in model-based SE | Type I error | Percentage increase in precision (ITT vs alternative) |
| --- | --- | --- | --- | --- | --- | --- |
| Intention to treat | -0.214 | 0.229 | 0.228 | -0.6 | 5.0 |  |
| Per protocol | -0.232 | 0.274 | 0.271 | -1.3 | 4.3 | 42.9 |
| IPW (all) | -0.232 | 0.290 | 0.296 | 1.9 | 4.2 | 60.2 |
| IPW (4993) | -0.231 | 0.290 | 0.296 | 2.2 | 4.2 | 59.3 |
| IV(Bayes) centred precise prior | -0.307 | 0.329 | 0.339 | 3.0 | 1.9 | 105.8 |
| IV(Bayes) centred vague prior | -0.308 | 0.329 | 0.384 | 16.6 | 1.3 | 106.0 |
| IV(Bayes) miscentred precise prior | -0.301 | 0.339 | 0.339 | -0.1 | 2.4 | 118.6 |
| IV(Bayes) miscentred vague prior | -0.301 | 0.339 | 0.384 | 13.0 | 1.4 | 118.9 |
| IV(interaction) (all) | -0.334 | 7.392 | 2296.663 | 30968.6 | 0.8 | 103780.1 |
| SE <= 100 SE(ITT) (4828) | -0.307 | 0.611 | 2.748 | 350.0 | 0.8 | 608.6 |
| SE <= 10 SE(ITT) (4369) | -0.306 | 0.416 | 0.689 | 65.5 | 0.9 | 229.3 |
| p < 0.05 (1003) | -0.306 | 0.358 | 0.367 | 2.4 | 2.1 | 143.9 |

**Scenario A; compliance scenario 4a**

| Analysis method | Mean of estimates | Empirical SE | Model-based SE | % bias in model-based SE | Type I error | Percentage increase in precision (ITT vs alternative) |
| --- | --- | --- | --- | --- | --- | --- |
| Intention to treat | -0.161 | 0.229 | 0.227 | -0.5 | 9.1 |  |
| Per protocol | -0.294 | 0.271 | 0.271 | -0.1 | 3.0 | 41.1 |
| IPW (all) | -0.292 | 0.289 | 0.296 | 2.6 | 2.6 | 59.9 |
| IPW (4983) | -0.292 | 0.287 | 0.296 | 3.2 | 2.6 | 58.0 |
| IV(Bayes) centred precise prior | -0.294 | 0.303 | 0.319 | 5.2 | 1.9 | 75.6 |
| IV(Bayes) centred vague prior | -0.294 | 0.303 | 0.394 | 30.1 | 1.1 | 76.0 |
| IV(Bayes) miscentred precise prior | -0.369 | 0.313 | 0.319 | 1.9 | 1.3 | 87.4 |
| IV(Bayes) miscentred vague prior | -0.369 | 0.313 | 0.394 | 26.1 | 0.8 | 87.4 |
| IV(interaction) (all) | -0.176 | 11.481 | 10027.985 | 87245.5 | 0.4 | 252316.7 |
| SE <= 100 SE(ITT) (4592) | -0.286 | 0.951 | 3.944 | 314.7 | 0.5 | 1631.6 |
| SE <= 10 SE(ITT) (3613) | -0.282 | 0.508 | 0.933 | 83.6 | 0.6 | 394.0 |
| p < 0.05 (263) | -0.281 | 0.374 | 0.402 | 7.6 | 1.5 | 167.4 |

**Scenario A; compliance scenario 4b**

| Analysis method | Mean of estimates | Empirical SE | Model-based SE | % bias in model-based SE | Type I error | Percentage increase in precision (ITT vs alternative) |
| --- | --- | --- | --- | --- | --- | --- |
| Intention to treat | -0.209 | 0.232 | 0.229 | -1.4 | 6.1 |  |
| Per protocol | -0.228 | 0.271 | 0.271 | -0.1 | 4.6 | 36.3 |
| IPW (all) | -0.228 | 0.286 | 0.296 | 3.4 | 4.0 | 51.9 |
| IPW (4996) | -0.228 | 0.285 | 0.296 | 3.6 | 4.0 | 51.3 |
| IV(Bayes) centred precise prior | -0.299 | 0.331 | 0.339 | 2.4 | 2.3 | 104.0 |
| IV(Bayes) centred vague prior | -0.299 | 0.332 | 0.385 | 16.1 | 1.5 | 104.3 |
| IV(Bayes) miscentred precise prior | -0.294 | 0.340 | 0.340 | -0.1 | 2.6 | 114.8 |
| IV(Bayes) miscentred vague prior | -0.294 | 0.340 | 0.385 | 13.3 | 1.7 | 114.4 |
| IV(interaction) (all) | -0.016 | 17.138 | 21698.553 | 126512.5 | 0.5 | 545976.6 |
| SE <= 100 SE(ITT) (4708) | -0.285 | 0.738 | 3.626 | 391.3 | 0.5 | 912.9 |
| SE <= 10 SE(ITT) (3888) | -0.301 | 0.448 | 0.861 | 92.1 | 0.6 | 273.6 |
| p < 0.05 (286) | -0.275 | 0.345 | 0.375 | 8.6 | 2.4 | 121.7 |

**Scenario B; compliance scenario 1**

| Analysis method | Mean of estimates | Empirical SE | Model-based SE | % bias in model-based SE | Type I error | Percentage increase in precision (ITT vs alternative) |
| --- | --- | --- | --- | --- | --- | --- |
| Intention to treat | -0.209 | 0.101 | 0.102 | 0.8 | 14.3 |  |
| Per protocol | -0.298 | 0.120 | 0.120 | 0.1 | 2.6 | 39.7 |
| IPW (all) | -0.299 | 0.128 | 0.131 | 2.2 | 2.3 | 60.9 |
| IPW (5000) | -0.299 | 0.128 | 0.131 | 2.2 | 2.3 | 60.9 |
| IV(Bayes) centred precise prior | -0.299 | 0.142 | 0.149 | 4.4 | 2.1 | 97.6 |
| IV(Bayes) centred vague prior | -0.299 | 0.143 | 0.168 | 18.0 | 1.4 | 98.4 |
| IV(Bayes) miscentred precise prior | -0.298 | 0.146 | 0.148 | 1.3 | 2.4 | 109.6 |
| IV(Bayes) miscentred vague prior | -0.298 | 0.147 | 0.168 | 14.6 | 1.5 | 110.4 |
| IV(interaction) (all) | -0.615 | 19.137 | 1026.747 | 5265.2 | 0.5 | 3577458.8 |
| SE <= 100 SE(ITT) (4730) | -0.295 | 0.334 | 1.564 | 368.3 | 0.6 | 989.1 |
| SE <= 10 SE(ITT) (3906) | -0.300 | 0.194 | 0.382 | 96.8 | 0.7 | 268.5 |
| p < 0.05 (255) | -0.314 | 0.158 | 0.173 | 9.4 | 1.2 | 144.3 |

**Scenario B; compliance scenario 2a**

| Analysis method | Mean of estimates | Empirical SE | Model-based SE | % bias in model-based SE | Type I error | Percentage increase in precision (ITT vs alternative) |
| --- | --- | --- | --- | --- | --- | --- |
| Intention to treat | -0.165 | 0.104 | 0.102 | -2.1 | 26.6 |  |
| Per protocol | -0.299 | 0.121 | 0.120 | -0.6 | 2.7 | 34.6 |
| IPW (all) | -0.299 | 0.128 | 0.132 | 2.7 | 2.4 | 51.8 |
| IPW (5000) | -0.299 | 0.128 | 0.132 | 2.7 | 2.4 | 51.8 |
| IV(Bayes) centred precise prior | -0.299 | 0.137 | 0.151 | 10.1 | 1.7 | 72.3 |
| IV(Bayes) centred vague prior | -0.300 | 0.138 | 0.242 | 75.6 | 0.3 | 74.4 |
| IV(Bayes) miscentred precise prior | -0.378 | 0.140 | 0.150 | 7.2 | 0.5 | 81.4 |
| IV(Bayes) miscentred vague prior | -0.378 | 0.141 | 0.242 | 71.8 | 0.2 | 82.0 |
| IV(interaction) (all) | -0.936 | 52.968 | 6655.385 | 12464.9 | 0.2 | 2.6e+07 |
| SE <= 100 SE(ITT) (4449) | -0.314 | 0.630 | 2.192 | 247.9 | 0.2 | 3556.3 |
| SE <= 10 SE(ITT) (3009) | -0.304 | 0.309 | 0.519 | 67.8 | 0.3 | 780.7 |
| p < 0.05 (275) | -0.306 | 0.227 | 0.241 | 6.2 | 1.5 | 373.1 |

**Scenario B; compliance scenario 2b**

| Analysis method | Mean of estimates | Empirical SE | Model-based SE | % bias in model-based SE | Type I error | Percentage increase in precision (ITT vs alternative) |
| --- | --- | --- | --- | --- | --- | --- |
| Intention to treat | -0.211 | 0.103 | 0.102 | -0.7 | 14.1 |  |
| Per protocol | -0.301 | 0.119 | 0.120 | 0.7 | 2.0 | 34.8 |
| IPW (all) | -0.300 | 0.128 | 0.131 | 3.0 | 1.9 | 54.5 |
| IPW (5000) | -0.300 | 0.128 | 0.131 | 3.0 | 1.9 | 54.5 |
| IV(Bayes) centred precise prior | -0.301 | 0.144 | 0.148 | 3.1 | 2.1 | 96.8 |
| IV(Bayes) centred vague prior | -0.301 | 0.144 | 0.168 | 16.2 | 1.6 | 97.7 |
| IV(Bayes) miscentred precise prior | -0.300 | 0.148 | 0.148 | 0.3 | 2.4 | 107.7 |
| IV(Bayes) miscentred vague prior | -0.300 | 0.148 | 0.168 | 13.2 | 1.7 | 108.6 |
| IV(interaction) (all) | -0.298 | 0.388 | 13.402 | 3354.3 | 1.5 | 1327.6 |
| SE <= 100 SE(ITT) (4984) | -0.302 | 0.172 | 0.357 | 107.3 | 1.5 | 180.5 |
| SE <= 10 SE(ITT) (4935) | -0.302 | 0.161 | 0.196 | 22.0 | 1.5 | 145.4 |
| p < 0.05 (3443) | -0.302 | 0.152 | 0.161 | 6.4 | 1.8 | 118.1 |

**Scenario B; compliance scenario 2c**

| Analysis method | Mean of estimates | Empirical SE | Model-based SE | % bias in model-based SE | Type I error | Percentage increase in precision (ITT vs alternative) |
| --- | --- | --- | --- | --- | --- | --- |
| Intention to treat | -0.165 | 0.102 | 0.102 | -0.2 | 25.9 |  |
| Per protocol | -0.301 | 0.120 | 0.120 | 0.6 | 2.5 | 37.1 |
| IPW (all) | -0.301 | 0.128 | 0.132 | 3.2 | 2.3 | 56.0 |
| IPW (5000) | -0.301 | 0.128 | 0.132 | 3.2 | 2.3 | 56.0 |
| IV(Bayes) centred precise prior | -0.299 | 0.133 | 0.150 | 12.9 | 1.6 | 69.9 |
| IV(Bayes) centred vague prior | -0.299 | 0.134 | 0.242 | 80.7 | 0.3 | 72.2 |
| IV(Bayes) miscentred precise prior | -0.378 | 0.136 | 0.150 | 10.4 | 0.6 | 77.6 |
| IV(Bayes) miscentred vague prior | -0.378 | 0.137 | 0.242 | 77.3 | 0.1 | 78.9 |
| IV(interaction) (all) | -0.298 | 0.935 | 80.172 | 8470.6 | 1.1 | 8268.4 |
| SE <= 100 SE(ITT) (4965) | -0.300 | 0.287 | 0.604 | 110.7 | 1.1 | 685.4 |
| SE <= 10 SE(ITT) (4833) | -0.301 | 0.231 | 0.284 | 22.8 | 1.1 | 411.5 |
| p < 0.05 (3379) | -0.303 | 0.198 | 0.210 | 6.4 | 1.5 | 273.9 |

**Scenario B; compliance scenario 3a**

| Analysis method | Mean of estimates | Empirical SE | Model-based SE | % bias in model-based SE | Type I error | Percentage increase in precision (ITT vs alternative) |
| --- | --- | --- | --- | --- | --- | --- |
| Intention to treat | -0.166 | 0.101 | 0.101 | 0.4 | 26.3 |  |
| Per protocol | -0.297 | 0.120 | 0.120 | 0.1 | 2.7 | 41.8 |
| IPW (all) | -0.297 | 0.128 | 0.132 | 3.1 | 2.2 | 60.9 |
| IPW (5000) | -0.297 | 0.128 | 0.132 | 3.1 | 2.2 | 60.9 |
| IV(Bayes) centred precise prior | -0.301 | 0.134 | 0.149 | 11.8 | 1.5 | 76.1 |
| IV(Bayes) centred vague prior | -0.301 | 0.134 | 0.242 | 80.5 | 0.2 | 77.0 |
| IV(Bayes) miscentred precise prior | -0.381 | 0.139 | 0.149 | 7.7 | 0.4 | 89.6 |
| IV(Bayes) miscentred vague prior | -0.381 | 0.139 | 0.242 | 73.8 | 0.1 | 90.6 |
| IV(interaction) (all) | -0.626 | 13.779 | 9809.587 | 71092.6 | 0.1 | 1870234.1 |
| SE <= 100 SE(ITT) (4412) | -0.278 | 0.637 | 2.220 | 248.4 | 0.1 | 3897.5 |
| SE <= 10 SE(ITT) (2968) | -0.280 | 0.291 | 0.502 | 72.6 | 0.2 | 731.7 |
| p < 0.05 (271) | -0.292 | 0.194 | 0.237 | 22.0 | 0.0 | 272.1 |

**Scenario B; compliance scenario 3b**

| Analysis method | Mean of estimates | Empirical SE | Model-based SE | % bias in model-based SE | Type I error | Percentage increase in precision (ITT vs alternative) |
| --- | --- | --- | --- | --- | --- | --- |
| Intention to treat | -0.209 | 0.102 | 0.102 | 0.2 | 14.4 |  |
| Per protocol | -0.228 | 0.120 | 0.120 | 0.3 | 8.0 | 38.9 |
| IPW (all) | -0.229 | 0.128 | 0.131 | 3.0 | 7.2 | 57.8 |
| IPW (5000) | -0.229 | 0.128 | 0.131 | 3.0 | 7.2 | 57.8 |
| IV(Bayes) centred precise prior | -0.299 | 0.143 | 0.148 | 3.2 | 2.2 | 99.3 |
| IV(Bayes) centred vague prior | -0.299 | 0.144 | 0.168 | 16.7 | 1.6 | 100.1 |
| IV(Bayes) miscentred precise prior | -0.298 | 0.148 | 0.148 | -0.1 | 2.5 | 112.6 |
| IV(Bayes) miscentred vague prior | -0.298 | 0.148 | 0.168 | 13.3 | 1.8 | 112.4 |
| IV(interaction) (all) | -0.298 | 0.290 | 43.345 | 14861.2 | 1.5 | 714.6 |
| SE <= 100 SE(ITT) (4987) | -0.299 | 0.179 | 0.447 | 150.3 | 1.5 | 209.6 |
| SE <= 10 SE(ITT) (4931) | -0.298 | 0.162 | 0.195 | 20.8 | 1.5 | 154.1 |
| p < 0.05 (3434) | -0.301 | 0.152 | 0.161 | 6.1 | 1.9 | 123.9 |

**Scenario B; compliance scenario 4a**

| Analysis method | Mean of estimates | Empirical SE | Model-based SE | % bias in model-based SE | Type I error | Percentage increase in precision (ITT vs alternative) |
| --- | --- | --- | --- | --- | --- | --- |
| Intention to treat | -0.167 | 0.102 | 0.101 | -0.4 | 26.8 |  |
| Per protocol | -0.296 | 0.120 | 0.120 | 0.3 | 2.7 | 38.9 |
| IPW (all) | -0.296 | 0.127 | 0.131 | 3.4 | 2.2 | 56.7 |
| IPW (5000) | -0.296 | 0.127 | 0.131 | 3.4 | 2.2 | 56.7 |
| IV(Bayes) centred precise prior | -0.302 | 0.135 | 0.149 | 10.6 | 1.6 | 76.6 |
| IV(Bayes) centred vague prior | -0.301 | 0.135 | 0.240 | 77.6 | 0.4 | 77.7 |
| IV(Bayes) miscentred precise prior | -0.380 | 0.140 | 0.149 | 6.7 | 0.6 | 90.0 |
| IV(Bayes) miscentred vague prior | -0.380 | 0.141 | 0.240 | 70.8 | 0.3 | 91.9 |
| IV(interaction) (all) | -0.219 | 13.427 | 7075.730 | 52599.2 | 0.1 | 1747858.4 |
| SE <= 100 SE(ITT) (4405) | -0.280 | 0.619 | 2.185 | 252.7 | 0.1 | 3620.5 |
| SE <= 10 SE(ITT) (2944) | -0.279 | 0.299 | 0.513 | 72.0 | 0.2 | 764.2 |
| p < 0.05 (246) | -0.296 | 0.209 | 0.237 | 13.9 | 0.8 | 321.6 |

**Scenario B; compliance scenario 4b**

| Analysis method | Mean of estimates | Empirical SE | Model-based SE | % bias in model-based SE | Type I error | Percentage increase in precision (ITT vs alternative) |
| --- | --- | --- | --- | --- | --- | --- |
| Intention to treat | -0.207 | 0.103 | 0.102 | -0.8 | 15.1 |  |
| Per protocol | -0.226 | 0.119 | 0.120 | 0.2 | 8.3 | 35.7 |
| IPW (all) | -0.227 | 0.128 | 0.131 | 2.6 | 7.3 | 55.0 |
| IPW (5000) | -0.227 | 0.128 | 0.131 | 2.6 | 7.3 | 55.0 |
| IV(Bayes) centred precise prior | -0.297 | 0.145 | 0.148 | 2.2 | 2.3 | 99.4 |
| IV(Bayes) centred vague prior | -0.297 | 0.145 | 0.168 | 16.0 | 1.5 | 99.2 |
| IV(Bayes) miscentred precise prior | -0.297 | 0.149 | 0.148 | -1.0 | 2.7 | 112.1 |
| IV(Bayes) miscentred vague prior | -0.297 | 0.150 | 0.168 | 12.2 | 1.7 | 112.7 |
| IV(interaction) (all) | -0.004 | 14.473 | 1208.167 | 8247.5 | 0.3 | 1992232.5 |
| SE <= 100 SE(ITT) (4685) | -0.292 | 0.329 | 1.653 | 403.0 | 0.4 | 926.7 |
| SE <= 10 SE(ITT) (3858) | -0.293 | 0.195 | 0.382 | 95.4 | 0.4 | 263.4 |
| p < 0.05 (260) | -0.294 | 0.142 | 0.169 | 18.5 | 0.0 | 92.8 |

**Scenario C; compliance scenario 1**

| Analysis method | Mean of estimates | Empirical SE | Model-based SE | % bias in model-based SE | Type I error | Percentage increase in precision (ITT vs alternative) |
| --- | --- | --- | --- | --- | --- | --- |
| Intention to treat | -0.271 | 0.100 | 0.101 | 0.4 | 4.8 |  |
| Per protocol | -0.302 | 0.104 | 0.106 | 1.3 | 2.1 | 7.5 |
| IPW (all) | -0.301 | 0.114 | 0.116 | 1.2 | 2.6 | 29.1 |
| IPW (5000) | -0.301 | 0.114 | 0.116 | 1.2 | 2.6 | 29.1 |
| IV(Bayes) centred precise prior | -0.301 | 0.111 | 0.113 | 2.0 | 2.2 | 21.7 |
| IV(Bayes) centred vague prior | -0.302 | 0.111 | 0.120 | 8.3 | 2.0 | 21.8 |
| IV(Bayes) miscentred precise prior | -0.302 | 0.112 | 0.113 | 0.9 | 2.3 | 24.1 |
| IV(Bayes) miscentred vague prior | -0.302 | 0.112 | 0.120 | 7.2 | 1.8 | 24.2 |
| IV(interaction) (all) | -0.211 | 4.758 | 1045.193 | 21865.8 | 0.4 | 224113.7 |
| SE <= 100 SE(ITT) (4735) | -0.300 | 0.279 | 1.531 | 449.0 | 0.4 | 670.4 |
| SE <= 10 SE(ITT) (4005) | -0.304 | 0.160 | 0.341 | 113.0 | 0.4 | 153.7 |
| p < 0.05 (237) | -0.308 | 0.109 | 0.132 | 21.4 | 0.8 | 16.9 |

**Scenario C; compliance scenario 2a**

| Analysis method | Mean of estimates | Empirical SE | Model-based SE | % bias in model-based SE | Type I error | Percentage increase in precision (ITT vs alternative) |
| --- | --- | --- | --- | --- | --- | --- |
| Intention to treat | -0.247 | 0.100 | 0.101 | 0.8 | 7.3 |  |
| Per protocol | -0.301 | 0.104 | 0.106 | 1.3 | 2.3 | 8.2 |
| IPW (all) | -0.300 | 0.116 | 0.116 | -0.1 | 2.7 | 33.4 |
| IPW (4994) | -0.301 | 0.114 | 0.116 | 1.2 | 2.5 | 30.2 |
| IV(Bayes) centred precise prior | -0.300 | 0.108 | 0.113 | 4.8 | 2.0 | 15.2 |
| IV(Bayes) centred vague prior | -0.300 | 0.108 | 0.139 | 29.2 | 0.9 | 15.2 |
| IV(Bayes) miscentred precise prior | -0.332 | 0.109 | 0.113 | 3.7 | 1.0 | 17.4 |
| IV(Bayes) miscentred vague prior | -0.332 | 0.109 | 0.139 | 27.7 | 0.6 | 17.7 |
| IV(interaction) (all) | -0.277 | 12.970 | 2482.909 | 19043.4 | 0.2 | 1675662.0 |
| SE <= 100 SE(ITT) (4526) | -0.304 | 0.440 | 1.832 | 316.2 | 0.2 | 1829.9 |
| SE <= 10 SE(ITT) (3459) | -0.306 | 0.227 | 0.424 | 87.0 | 0.2 | 412.5 |
| p < 0.05 (261) | -0.308 | 0.143 | 0.164 | 15.1 | 1.1 | 102.3 |

**Scenario C; compliance scenario 2b**

| Analysis method | Mean of estimates | Empirical SE | Model-based SE | % bias in model-based SE | Type I error | Percentage increase in precision (ITT vs alternative) |
| --- | --- | --- | --- | --- | --- | --- |
| Intention to treat | -0.266 | 0.100 | 0.101 | 0.9 | 5.2 |  |
| Per protocol | -0.296 | 0.106 | 0.106 | -0.1 | 2.8 | 12.0 |
| IPW (all) | -0.299 | 0.121 | 0.116 | -4.2 | 2.8 | 46.1 |
| IPW (4978) | -0.296 | 0.115 | 0.116 | 0.8 | 2.8 | 31.8 |
| IV(Bayes) centred precise prior | -0.295 | 0.111 | 0.113 | 2.3 | 2.4 | 22.3 |
| IV(Bayes) centred vague prior | -0.295 | 0.111 | 0.120 | 8.5 | 2.0 | 22.4 |
| IV(Bayes) miscentred precise prior | -0.295 | 0.112 | 0.113 | 1.0 | 2.5 | 25.4 |
| IV(Bayes) miscentred vague prior | -0.295 | 0.112 | 0.120 | 7.0 | 2.0 | 25.8 |
| IV(interaction) (all) | -0.294 | 0.115 | 0.121 | 6.1 | 2.0 | 31.0 |
| SE <= 100 SE(ITT) (5000) | -0.294 | 0.115 | 0.121 | 6.1 | 2.0 | 31.0 |
| SE <= 10 SE(ITT) (5000) | -0.294 | 0.115 | 0.121 | 6.1 | 2.0 | 31.0 |
| p < 0.05 (4847) | -0.295 | 0.114 | 0.120 | 5.4 | 2.0 | 29.1 |

**Scenario C; compliance scenario 2c**

| Analysis method | Mean of estimates | Empirical SE | Model-based SE | % bias in model-based SE | Type I error | Percentage increase in precision (ITT vs alternative) |
| --- | --- | --- | --- | --- | --- | --- |
| Intention to treat | -0.244 | 0.101 | 0.101 | -0.4 | 7.8 |  |
| Per protocol | -0.297 | 0.106 | 0.106 | -0.6 | 2.5 | 10.1 |
| IPW (all) | -0.317 | 0.154 | 0.117 | -24.2 | 2.3 | 130.1 |
| IPW (4794) | -0.296 | 0.116 | 0.116 | -0.2 | 2.4 | 30.8 |
| IV(Bayes) centred precise prior | -0.296 | 0.109 | 0.113 | 3.3 | 2.4 | 15.7 |
| IV(Bayes) centred vague prior | -0.297 | 0.109 | 0.139 | 27.7 | 1.1 | 15.5 |
| IV(Bayes) miscentred precise prior | -0.329 | 0.110 | 0.113 | 2.3 | 1.2 | 18.0 |
| IV(Bayes) miscentred vague prior | -0.329 | 0.110 | 0.139 | 26.0 | 0.8 | 18.6 |
| IV(interaction) (all) | -0.297 | 0.133 | 0.141 | 6.2 | 1.9 | 71.5 |
| SE <= 100 SE(ITT) (5000) | -0.297 | 0.133 | 0.141 | 6.2 | 1.9 | 71.5 |
| SE <= 10 SE(ITT) (5000) | -0.297 | 0.133 | 0.141 | 6.2 | 1.9 | 71.5 |
| p < 0.05 (4849) | -0.297 | 0.130 | 0.135 | 4.0 | 2.0 | 64.7 |

**Scenario C; compliance scenario 3a**

| Analysis method | Mean of estimates | Empirical SE | Model-based SE | % bias in model-based SE | Type I error | Percentage increase in precision (ITT vs alternative) |
| --- | --- | --- | --- | --- | --- | --- |
| Intention to treat | -0.248 | 0.100 | 0.101 | 0.6 | 7.7 |  |
| Per protocol | -0.300 | 0.106 | 0.106 | -0.1 | 2.6 | 11.3 |
| IPW (all) | -0.300 | 0.115 | 0.116 | 0.2 | 2.5 | 32.5 |
| IPW (5000) | -0.300 | 0.115 | 0.116 | 0.2 | 2.5 | 32.5 |
| IV(Bayes) centred precise prior | -0.301 | 0.108 | 0.112 | 4.5 | 2.2 | 15.4 |
| IV(Bayes) centred vague prior | -0.301 | 0.108 | 0.139 | 28.9 | 1.0 | 15.7 |
| IV(Bayes) miscentred precise prior | -0.333 | 0.109 | 0.112 | 3.3 | 1.1 | 18.1 |
| IV(Bayes) miscentred vague prior | -0.333 | 0.109 | 0.139 | 27.4 | 0.6 | 18.6 |
| IV(interaction) (all) | 0.089 | 18.007 | 4047.873 | 22379.8 | 0.2 | 3229877.0 |
| SE <= 100 SE(ITT) (4507) | -0.295 | 0.453 | 1.880 | 315.1 | 0.2 | 1943.2 |
| SE <= 10 SE(ITT) (3424) | -0.291 | 0.224 | 0.427 | 90.8 | 0.2 | 399.3 |
| p < 0.05 (252) | -0.297 | 0.138 | 0.164 | 18.5 | 0.8 | 91.1 |

**Scenario C; compliance scenario 3b**

| Analysis method | Mean of estimates | Empirical SE | Model-based SE | % bias in model-based SE | Type I error | Percentage increase in precision (ITT vs alternative) |
| --- | --- | --- | --- | --- | --- | --- |
| Intention to treat | -0.269 | 0.103 | 0.101 | -2.5 | 5.6 |  |
| Per protocol | -0.272 | 0.108 | 0.106 | -2.5 | 5.0 | 9.8 |
| IPW (all) | -0.272 | 0.119 | 0.116 | -2.6 | 4.6 | 31.6 |
| IPW (4996) | -0.272 | 0.118 | 0.116 | -1.8 | 4.6 | 29.6 |
| IV(Bayes) centred precise prior | -0.299 | 0.114 | 0.113 | -1.3 | 2.9 | 22.5 |
| IV(Bayes) centred vague prior | -0.299 | 0.114 | 0.120 | 4.9 | 2.3 | 22.4 |
| IV(Bayes) miscentred precise prior | -0.299 | 0.116 | 0.113 | -2.6 | 3.0 | 26.0 |
| IV(Bayes) miscentred vague prior | -0.299 | 0.116 | 0.120 | 3.5 | 2.3 | 25.8 |
| IV(interaction) (all) | -0.288 | 0.784 | 350.282 | 44585.2 | 1.4 | 5650.6 |
| SE <= 100 SE(ITT) (4958) | -0.303 | 0.164 | 0.681 | 315.6 | 1.4 | 151.4 |
| SE <= 10 SE(ITT) (4836) | -0.301 | 0.140 | 0.202 | 44.2 | 1.4 | 82.9 |
| p < 0.05 (2327) | -0.299 | 0.122 | 0.128 | 5.1 | 2.0 | 39.1 |

**Scenario C; compliance scenario 4a**

| Analysis method | Mean of estimates | Empirical SE | Model-based SE | % bias in model-based SE | Type I error | Percentage increase in precision (ITT vs alternative) |
| --- | --- | --- | --- | --- | --- | --- |
| Intention to treat | -0.248 | 0.101 | 0.101 | 0.1 | 7.4 |  |
| Per protocol | -0.301 | 0.106 | 0.106 | 0.0 | 2.1 | 10.4 |
| IPW (all) | -0.301 | 0.114 | 0.116 | 1.1 | 2.1 | 29.5 |
| IPW (5000) | -0.301 | 0.114 | 0.116 | 1.1 | 2.1 | 29.5 |
| IV(Bayes) centred precise prior | -0.301 | 0.108 | 0.112 | 3.6 | 2.1 | 16.2 |
| IV(Bayes) centred vague prior | -0.301 | 0.109 | 0.139 | 27.7 | 1.0 | 16.5 |
| IV(Bayes) miscentred precise prior | -0.333 | 0.110 | 0.112 | 2.2 | 1.2 | 19.6 |
| IV(Bayes) miscentred vague prior | -0.333 | 0.110 | 0.139 | 25.9 | 0.7 | 19.8 |
| IV(interaction) (all) | -3.756 | 216.430 | 2032.435 | 839.1 | 0.2 | 4.6e+08 |
| SE <= 100 SE(ITT) (4546) | -0.299 | 0.467 | 1.962 | 320.5 | 0.2 | 2053.3 |
| SE <= 10 SE(ITT) (3410) | -0.299 | 0.226 | 0.429 | 90.1 | 0.3 | 404.3 |
| p < 0.05 (263) | -0.286 | 0.150 | 0.162 | 8.0 | 1.9 | 123.7 |

**Scenario C; compliance scenario 4b**

| Analysis method | Mean of estimates | Empirical SE | Model-based SE | % bias in model-based SE | Type I error | Percentage increase in precision (ITT vs alternative) |
| --- | --- | --- | --- | --- | --- | --- |
| Intention to treat | -0.270 | 0.100 | 0.101 | 1.0 | 4.4 |  |
| Per protocol | -0.244 | 0.104 | 0.105 | 1.4 | 7.4 | 9.1 |
| IPW (all) | -0.245 | 0.113 | 0.115 | 2.3 | 6.2 | 28.2 |
| IPW (5000) | -0.245 | 0.113 | 0.115 | 2.3 | 6.2 | 28.2 |
| IV(Bayes) centred precise prior | -0.300 | 0.110 | 0.113 | 2.1 | 2.3 | 22.7 |
| IV(Bayes) centred vague prior | -0.301 | 0.111 | 0.120 | 8.2 | 1.9 | 23.2 |
| IV(Bayes) miscentred precise prior | -0.301 | 0.112 | 0.113 | 0.7 | 2.4 | 26.2 |
| IV(Bayes) miscentred vague prior | -0.301 | 0.112 | 0.120 | 6.8 | 2.0 | 26.5 |
| IV(interaction) (all) | -0.328 | 2.304 | 720.844 | 31184.8 | 0.2 | 53403.2 |
| SE <= 100 SE(ITT) (4716) | -0.291 | 0.268 | 1.501 | 459.3 | 0.3 | 626.2 |
| SE <= 10 SE(ITT) (4042) | -0.299 | 0.157 | 0.346 | 120.1 | 0.3 | 148.4 |
| p < 0.05 (217) | -0.286 | 0.123 | 0.132 | 7.0 | 0.9 | 53.4 |

**Scenario D; compliance scenario 1**

| Analysis method | Mean of estimates | Empirical SE | Model-based SE | % bias in model-based SE | Type I error | Percentage increase in precision (ITT vs alternative) |
| --- | --- | --- | --- | --- | --- | --- |
| Intention to treat | -0.210 | 0.206 | 0.207 | 0.1 | 6.2 |  |
| Per protocol | -0.298 | 0.244 | 0.243 | -0.4 | 2.4 | 39.5 |
| IPW (all) | -0.298 | 0.244 | 0.244 | -0.0 | 2.4 | 39.3 |
| IPW (4994) | -0.298 | 0.244 | 0.244 | 0.0 | 2.4 | 39.1 |
| IV(Bayes) centred precise prior | -0.301 | 0.292 | 0.309 | 5.7 | 1.9 | 100.0 |
| IV(Bayes) centred vague prior | -0.301 | 0.292 | 0.359 | 22.8 | 1.2 | 100.5 |
| IV(Bayes) miscentred precise prior | -0.295 | 0.303 | 0.309 | 2.0 | 2.2 | 114.7 |
| IV(Bayes) miscentred vague prior | -0.295 | 0.302 | 0.359 | 18.7 | 1.3 | 114.5 |
| IV(interaction) (all) | -0.357 | 6.329 | 3416.906 | 53889.4 | 0.3 | 93844.5 |
| SE <= 100 SE(ITT) (4730) | -0.309 | 0.654 | 3.172 | 385.2 | 0.3 | 902.3 |
| SE <= 10 SE(ITT) (3909) | -0.308 | 0.396 | 0.775 | 95.7 | 0.3 | 267.9 |
| p < 0.05 (254) | -0.308 | 0.303 | 0.332 | 9.4 | 0.4 | 115.4 |

**Scenario D; compliance scenario 2a**

| Analysis method | Mean of estimates | Empirical SE | Model-based SE | % bias in model-based SE | Type I error | Percentage increase in precision (ITT vs alternative) |
| --- | --- | --- | --- | --- | --- | --- |
| Intention to treat | -0.164 | 0.209 | 0.207 | -1.3 | 10.0 |  |
| Per protocol | -0.301 | 0.248 | 0.243 | -1.8 | 2.7 | 39.8 |
| IPW (all) | -0.299 | 0.248 | 0.245 | -1.5 | 2.7 | 40.4 |
| IPW (4961) | -0.301 | 0.247 | 0.244 | -1.3 | 2.7 | 39.5 |
| IV(Bayes) centred precise prior | -0.299 | 0.276 | 0.291 | 5.4 | 2.0 | 73.7 |
| IV(Bayes) centred vague prior | -0.299 | 0.276 | 0.372 | 34.7 | 1.2 | 73.7 |
| IV(Bayes) miscentred precise prior | -0.376 | 0.285 | 0.291 | 2.1 | 1.7 | 85.0 |
| IV(Bayes) miscentred vague prior | -0.376 | 0.286 | 0.372 | 29.9 | 0.9 | 86.7 |
| IV(interaction) (all) | -0.175 | 8.362 | 3692.287 | 44054.6 | 0.3 | 159238.3 |
| SE <= 100 SE(ITT) (4593) | -0.275 | 0.824 | 3.596 | 336.3 | 0.3 | 1447.7 |
| SE <= 10 SE(ITT) (3611) | -0.298 | 0.454 | 0.865 | 90.5 | 0.4 | 369.7 |
| p < 0.05 (275) | -0.341 | 0.351 | 0.359 | 2.3 | 1.5 | 181.2 |

**Scenario D; compliance scenario 2b**

| Analysis method | Mean of estimates | Empirical SE | Model-based SE | % bias in model-based SE | Type I error | Percentage increase in precision (ITT vs alternative) |
| --- | --- | --- | --- | --- | --- | --- |
| Intention to treat | -0.212 | 0.207 | 0.207 | 0.1 | 6.4 |  |
| Per protocol | -0.298 | 0.243 | 0.244 | 0.5 | 2.6 | 38.4 |
| IPW (all) | -0.298 | 0.244 | 0.245 | 0.1 | 2.6 | 39.8 |
| IPW (4989) | -0.298 | 0.244 | 0.244 | 0.1 | 2.6 | 39.6 |
| IV(Bayes) centred precise prior | -0.303 | 0.294 | 0.309 | 5.4 | 2.1 | 101.7 |
| IV(Bayes) centred vague prior | -0.302 | 0.294 | 0.360 | 22.6 | 1.5 | 102.2 |
| IV(Bayes) miscentred precise prior | -0.295 | 0.304 | 0.310 | 1.8 | 2.7 | 116.4 |
| IV(Bayes) miscentred vague prior | -0.295 | 0.304 | 0.360 | 18.6 | 1.5 | 116.0 |
| IV(interaction) (all) | -0.045 | 7.275 | 4774.464 | 65528.4 | 0.8 | 123794.5 |
| SE <= 100 SE(ITT) (4821) | -0.302 | 0.549 | 2.319 | 322.6 | 0.9 | 604.8 |
| SE <= 10 SE(ITT) (4369) | -0.301 | 0.378 | 0.634 | 67.8 | 0.9 | 233.9 |
| p < 0.05 (1002) | -0.283 | 0.316 | 0.334 | 5.5 | 2.1 | 134.4 |

**Scenario D; compliance scenario 2c**

| Analysis method | Mean of estimates | Empirical SE | Model-based SE | % bias in model-based SE | Type I error | Percentage increase in precision (ITT vs alternative) |
| --- | --- | --- | --- | --- | --- | --- |
| Intention to treat | -0.167 | 0.206 | 0.207 | 0.6 | 9.0 |  |
| Per protocol | -0.303 | 0.243 | 0.244 | 0.7 | 2.1 | 39.2 |
| IPW (all) | -0.303 | 0.244 | 0.245 | 0.4 | 2.2 | 40.8 |
| IPW (4970) | -0.302 | 0.243 | 0.245 | 0.6 | 2.2 | 40.1 |
| IV(Bayes) centred precise prior | -0.303 | 0.270 | 0.291 | 7.9 | 1.6 | 72.2 |
| IV(Bayes) centred vague prior | -0.303 | 0.270 | 0.372 | 37.9 | 0.6 | 72.4 |
| IV(Bayes) miscentred precise prior | -0.381 | 0.278 | 0.291 | 4.8 | 0.9 | 82.3 |
| IV(Bayes) miscentred vague prior | -0.381 | 0.278 | 0.372 | 33.8 | 0.4 | 82.8 |
| IV(interaction) (all) | -0.504 | 9.100 | 3712.802 | 40700.9 | 0.5 | 195623.8 |
| SE <= 100 SE(ITT) (4768) | -0.302 | 0.697 | 2.696 | 287.0 | 0.5 | 1046.9 |
| SE <= 10 SE(ITT) (4197) | -0.305 | 0.424 | 0.697 | 64.5 | 0.5 | 325.0 |
| p < 0.05 (1002) | -0.300 | 0.336 | 0.362 | 7.7 | 1.4 | 167.4 |

**Scenario D; compliance scenario 3a**

| Analysis method | Mean of estimates | Empirical SE | Model-based SE | % bias in model-based SE | Type I error | Percentage increase in precision (ITT vs alternative) |
| --- | --- | --- | --- | --- | --- | --- |
| Intention to treat | -0.164 | 0.209 | 0.207 | -1.1 | 9.5 |  |
| Per protocol | -0.297 | 0.242 | 0.244 | 0.7 | 2.7 | 34.4 |
| IPW (all) | -0.295 | 0.244 | 0.246 | 0.7 | 2.7 | 35.9 |
| IPW (4983) | -0.296 | 0.244 | 0.245 | 0.7 | 2.7 | 35.7 |
| IV(Bayes) centred precise prior | -0.298 | 0.276 | 0.292 | 5.6 | 2.1 | 74.4 |
| IV(Bayes) centred vague prior | -0.298 | 0.277 | 0.370 | 33.9 | 1.0 | 74.8 |
| IV(Bayes) miscentred precise prior | -0.373 | 0.285 | 0.292 | 2.4 | 1.6 | 85.4 |
| IV(Bayes) miscentred vague prior | -0.373 | 0.285 | 0.370 | 29.9 | 0.8 | 85.7 |
| IV(interaction) (all) | -0.493 | 10.256 | 6885.866 | 67041.9 | 0.4 | 240331.1 |
| SE <= 100 SE(ITT) (4639) | -0.283 | 0.840 | 3.592 | 327.4 | 0.4 | 1514.3 |
| SE <= 10 SE(ITT) (3697) | -0.292 | 0.446 | 0.849 | 90.2 | 0.5 | 355.2 |
| p < 0.05 (306) | -0.286 | 0.319 | 0.367 | 15.2 | 1.6 | 131.9 |

**Scenario D; compliance scenario 3b**

| Analysis method | Mean of estimates | Empirical SE | Model-based SE | % bias in model-based SE | Type I error | Percentage increase in precision (ITT vs alternative) |
| --- | --- | --- | --- | --- | --- | --- |
| Intention to treat | -0.211 | 0.206 | 0.207 | 0.3 | 6.5 |  |
| Per protocol | -0.289 | 0.244 | 0.244 | -0.1 | 2.5 | 40.7 |
| IPW (all) | -0.289 | 0.245 | 0.245 | -0.2 | 2.8 | 41.9 |
| IPW (4987) | -0.288 | 0.245 | 0.245 | -0.2 | 2.8 | 41.6 |
| IV(Bayes) centred precise prior | -0.300 | 0.292 | 0.309 | 5.7 | 2.3 | 101.2 |
| IV(Bayes) centred vague prior | -0.300 | 0.292 | 0.359 | 22.8 | 1.3 | 101.4 |
| IV(Bayes) miscentred precise prior | -0.293 | 0.304 | 0.309 | 1.7 | 2.7 | 117.2 |
| IV(Bayes) miscentred vague prior | -0.293 | 0.304 | 0.359 | 18.2 | 1.5 | 117.2 |
| IV(interaction) (all) | -0.272 | 3.729 | 1080.890 | 28886.5 | 0.7 | 32676.7 |
| SE <= 100 SE(ITT) (4832) | -0.291 | 0.536 | 2.345 | 337.5 | 0.7 | 577.3 |
| SE <= 10 SE(ITT) (4394) | -0.298 | 0.374 | 0.636 | 69.9 | 0.8 | 230.5 |
| p < 0.05 (1000) | -0.302 | 0.312 | 0.334 | 6.8 | 1.8 | 130.2 |

**Scenario D; compliance scenario 4a**

| Analysis method | Mean of estimates | Empirical SE | Model-based SE | % bias in model-based SE | Type I error | Percentage increase in precision (ITT vs alternative) |
| --- | --- | --- | --- | --- | --- | --- |
| Intention to treat | -0.163 | 0.205 | 0.206 | 0.4 | 9.6 |  |
| Per protocol | -0.301 | 0.242 | 0.243 | 0.5 | 2.6 | 39.0 |
| IPW (all) | -0.301 | 0.242 | 0.244 | 0.8 | 2.5 | 39.0 |
| IPW (4986) | -0.301 | 0.242 | 0.244 | 0.7 | 2.5 | 39.1 |
| IV(Bayes) centred precise prior | -0.299 | 0.271 | 0.291 | 7.1 | 1.9 | 74.7 |
| IV(Bayes) centred vague prior | -0.298 | 0.272 | 0.375 | 37.9 | 1.1 | 75.1 |
| IV(Bayes) miscentred precise prior | -0.376 | 0.282 | 0.291 | 3.0 | 1.3 | 89.0 |
| IV(Bayes) miscentred vague prior | -0.376 | 0.283 | 0.374 | 32.5 | 0.8 | 89.5 |
| IV(interaction) (all) | -0.217 | 10.535 | 5458.443 | 51713.7 | 0.4 | 263036.1 |
| SE <= 100 SE(ITT) (4618) | -0.306 | 0.855 | 3.552 | 315.4 | 0.4 | 1634.3 |
| SE <= 10 SE(ITT) (3580) | -0.289 | 0.447 | 0.838 | 87.4 | 0.6 | 374.3 |
| p < 0.05 (267) | -0.277 | 0.338 | 0.369 | 9.0 | 1.9 | 171.5 |

**Scenario D; compliance scenario 4b**

| Analysis method | Mean of estimates | Empirical SE | Model-based SE | % bias in model-based SE | Type I error | Percentage increase in precision (ITT vs alternative) |
| --- | --- | --- | --- | --- | --- | --- |
| Intention to treat | -0.215 | 0.208 | 0.207 | -0.6 | 5.9 |  |
| Per protocol | -0.293 | 0.245 | 0.243 | -1.2 | 2.7 | 39.7 |
| IPW (all) | -0.293 | 0.245 | 0.243 | -1.0 | 2.7 | 39.6 |
| IPW (4988) | -0.293 | 0.246 | 0.243 | -1.1 | 2.7 | 39.7 |
| IV(Bayes) centred precise prior | -0.307 | 0.291 | 0.307 | 5.4 | 1.9 | 96.9 |
| IV(Bayes) centred vague prior | -0.307 | 0.292 | 0.355 | 21.8 | 1.3 | 97.1 |
| IV(Bayes) miscentred precise prior | -0.302 | 0.299 | 0.307 | 2.6 | 2.0 | 107.8 |
| IV(Bayes) miscentred vague prior | -0.302 | 0.300 | 0.355 | 18.6 | 1.4 | 108.1 |
| IV(interaction) (all) | -0.266 | 6.548 | 2527.267 | 38495.5 | 0.5 | 99270.2 |
| SE <= 100 SE(ITT) (4698) | -0.305 | 0.659 | 3.222 | 389.2 | 0.6 | 905.6 |
| SE <= 10 SE(ITT) (3923) | -0.301 | 0.395 | 0.762 | 93.1 | 0.7 | 261.2 |
| p < 0.05 (267) | -0.306 | 0.312 | 0.333 | 6.7 | 2.2 | 125.4 |

**Scenario E; compliance scenario 1**

| Analysis method | Mean of estimates | Empirical SE | Model-based SE | % bias in model-based SE | Power | Percentage increase in precision (ITT vs alternative) |
| --- | --- | --- | --- | --- | --- | --- |
| Intention to treat | 0.000 | 0.230 | 0.231 | 0.7 | 24.7 |  |
| Per protocol | -0.002 | 0.271 | 0.271 | -0.1 | 19.5 | 39.0 |
| IPW (all) | -0.002 | 0.286 | 0.296 | 3.2 | 16.3 | 55.5 |
| IPW (4995) | -0.002 | 0.286 | 0.296 | 3.4 | 16.3 | 54.9 |
| IV(Bayes) centred precise prior | -0.001 | 0.321 | 0.343 | 6.8 | 13.2 | 95.9 |
| IV(Bayes) centred vague prior | -0.001 | 0.322 | 0.389 | 20.7 | 9.7 | 96.3 |
| IV(Bayes) miscentred precise prior | 0.004 | 0.331 | 0.343 | 3.8 | 13.5 | 107.4 |
| IV(Bayes) miscentred vague prior | 0.004 | 0.331 | 0.388 | 17.2 | 9.7 | 108.2 |
| IV(interaction) (all) | -0.002 | 7.336 | 2913.797 | 39616.5 | 3.3 | 101931.3 |
| SE <= 100 SE(ITT) (4704) | 0.005 | 0.719 | 3.514 | 388.6 | 3.6 | 880.6 |
| SE <= 10 SE(ITT) (3919) | 0.004 | 0.438 | 0.853 | 94.7 | 4.3 | 264.2 |
| p < 0.05 (281) | 0.024 | 0.368 | 0.381 | 3.4 | 13.5 | 156.7 |

**Scenario E; compliance scenario 2a**

| Analysis method | Mean of estimates | Empirical SE | Model-based SE | % bias in model-based SE | Power | Percentage increase in precision (ITT vs alternative) |
| --- | --- | --- | --- | --- | --- | --- |
| Intention to treat | 0.059 | 0.224 | 0.231 | 3.6 | 32.9 |  |
| Per protocol | -0.003 | 0.263 | 0.271 | 3.2 | 18.1 | 38.0 |
| IPW (all) | -0.002 | 0.283 | 0.298 | 5.3 | 15.0 | 59.9 |
| IPW (4965) | -0.004 | 0.280 | 0.297 | 6.1 | 14.8 | 57.3 |
| IV(Bayes) centred precise prior | -0.002 | 0.292 | 0.324 | 10.9 | 13.4 | 70.3 |
| IV(Bayes) centred vague prior | -0.002 | 0.292 | 0.398 | 36.4 | 7.0 | 70.2 |
| IV(Bayes) miscentred precise prior | -0.079 | 0.299 | 0.324 | 8.2 | 8.7 | 79.1 |
| IV(Bayes) miscentred vague prior | -0.078 | 0.299 | 0.398 | 33.1 | 5.5 | 79.3 |
| IV(interaction) (all) | -0.140 | 12.680 | 8499.519 | 66928.5 | 2.1 | 321695.5 |
| SE <= 100 SE(ITT) (4611) | 0.008 | 0.924 | 3.909 | 323.1 | 2.3 | 1608.0 |
| SE <= 10 SE(ITT) (3636) | -0.002 | 0.498 | 0.908 | 82.5 | 2.9 | 395.9 |
| p < 0.05 (283) | -0.016 | 0.356 | 0.398 | 12.0 | 7.4 | 153.2 |

**Scenario E; compliance scenario 2b**

| Analysis method | Mean of estimates | Empirical SE | Model-based SE | % bias in model-based SE | Power | Percentage increase in precision (ITT vs alternative) |
| --- | --- | --- | --- | --- | --- | --- |
| Intention to treat | 0.003 | 0.232 | 0.231 | -0.2 | 25.8 |  |
| Per protocol | 0.002 | 0.271 | 0.271 | -0.1 | 19.4 | 36.9 |
| IPW (all) | 0.001 | 0.291 | 0.296 | 1.9 | 16.3 | 57.1 |
| IPW (4987) | 0.002 | 0.289 | 0.296 | 2.2 | 16.3 | 55.9 |
| IV(Bayes) centred precise prior | 0.005 | 0.325 | 0.344 | 5.9 | 13.9 | 96.1 |
| IV(Bayes) centred vague prior | 0.005 | 0.325 | 0.391 | 20.4 | 10.4 | 96.4 |
| IV(Bayes) miscentred precise prior | 0.011 | 0.335 | 0.344 | 2.4 | 14.9 | 109.4 |
| IV(Bayes) miscentred vague prior | 0.011 | 0.335 | 0.391 | 16.6 | 10.3 | 109.2 |
| IV(interaction) (all) | 0.079 | 5.385 | 2773.162 | 51402.6 | 5.6 | 53850.4 |
| SE <= 100 SE(ITT) (4833) | 0.012 | 0.620 | 2.817 | 354.3 | 5.8 | 615.5 |
| SE <= 10 SE(ITT) (4363) | 0.005 | 0.413 | 0.712 | 72.3 | 6.4 | 217.8 |
| p < 0.05 (981) | -0.002 | 0.329 | 0.372 | 13.2 | 10.8 | 101.0 |

**Scenario E; compliance scenario 2c**

| Analysis method | Mean of estimates | Empirical SE | Model-based SE | % bias in model-based SE | Power | Percentage increase in precision (ITT vs alternative) |
| --- | --- | --- | --- | --- | --- | --- |
| Intention to treat | 0.059 | 0.231 | 0.232 | 0.3 | 33.6 |  |
| Per protocol | -0.002 | 0.272 | 0.272 | 0.1 | 18.1 | 38.8 |
| IPW (all) | -0.004 | 0.291 | 0.298 | 2.2 | 15.9 | 59.1 |
| IPW (4965) | -0.002 | 0.288 | 0.297 | 3.4 | 16.0 | 55.1 |
| IV(Bayes) centred precise prior | -0.003 | 0.302 | 0.324 | 7.2 | 13.8 | 71.1 |
| IV(Bayes) centred vague prior | -0.003 | 0.302 | 0.400 | 32.3 | 7.9 | 71.3 |
| IV(Bayes) miscentred precise prior | -0.081 | 0.310 | 0.324 | 4.4 | 9.1 | 80.3 |
| IV(Bayes) miscentred vague prior | -0.081 | 0.310 | 0.400 | 28.8 | 6.2 | 80.5 |
| IV(interaction) (all) | 0.069 | 15.467 | 28275.656 | 182716.9 | 4.3 | 448427.0 |
| SE <= 100 SE(ITT) (4737) | 0.011 | 0.776 | 2.991 | 285.4 | 4.6 | 1029.5 |
| SE <= 10 SE(ITT) (4137) | 0.004 | 0.478 | 0.788 | 64.7 | 5.2 | 329.2 |
| p < 0.05 (983) | -0.002 | 0.372 | 0.400 | 7.4 | 9.4 | 159.8 |

**Scenario E; compliance scenario 3a**

| Analysis method | Mean of estimates | Empirical SE | Model-based SE | % bias in model-based SE | Power | Percentage increase in precision (ITT vs alternative) |
| --- | --- | --- | --- | --- | --- | --- |
| Intention to treat | 0.063 | 0.233 | 0.229 | -2.0 | 35.0 |  |
| Per protocol | 0.008 | 0.274 | 0.271 | -1.0 | 20.5 | 38.0 |
| IPW (all) | 0.009 | 0.295 | 0.297 | 0.8 | 17.4 | 60.0 |
| IPW (4968) | 0.005 | 0.291 | 0.297 | 2.2 | 17.0 | 55.4 |
| IV(Bayes) centred precise prior | 0.004 | 0.308 | 0.320 | 3.8 | 15.2 | 74.8 |
| IV(Bayes) centred vague prior | 0.004 | 0.308 | 0.395 | 28.2 | 8.4 | 74.7 |
| IV(Bayes) miscentred precise prior | -0.072 | 0.319 | 0.320 | 0.2 | 10.4 | 87.1 |
| IV(Bayes) miscentred vague prior | -0.073 | 0.320 | 0.395 | 23.5 | 6.9 | 88.0 |
| IV(interaction) (all) | 0.260 | 10.418 | 4320.206 | 41368.3 | 2.7 | 199429.8 |
| SE <= 100 SE(ITT) (4633) | 0.033 | 0.919 | 3.823 | 316.0 | 3.0 | 1452.4 |
| SE <= 10 SE(ITT) (3718) | 0.026 | 0.513 | 0.948 | 84.8 | 3.7 | 383.7 |
| p < 0.05 (261) | 0.012 | 0.374 | 0.417 | 11.5 | 9.2 | 156.5 |

**Scenario E; compliance scenario 3b**

| Analysis method | Mean of estimates | Empirical SE | Model-based SE | % bias in model-based SE | Power | Percentage increase in precision (ITT vs alternative) |
| --- | --- | --- | --- | --- | --- | --- |
| Intention to treat | -0.007 | 0.237 | 0.231 | -2.5 | 24.5 |  |
| Per protocol | 0.065 | 0.272 | 0.271 | -0.2 | 25.7 | 31.1 |
| IPW (all) | 0.064 | 0.294 | 0.296 | 0.8 | 22.6 | 53.5 |
| IPW (4981) | 0.066 | 0.292 | 0.296 | 1.5 | 22.6 | 51.0 |
| IV(Bayes) centred precise prior | -0.012 | 0.333 | 0.344 | 3.3 | 13.1 | 96.7 |
| IV(Bayes) centred vague prior | -0.012 | 0.333 | 0.390 | 17.0 | 10.1 | 97.0 |
| IV(Bayes) miscentred precise prior | -0.008 | 0.342 | 0.344 | 0.7 | 13.4 | 107.1 |
| IV(Bayes) miscentred vague prior | -0.007 | 0.342 | 0.390 | 14.2 | 9.9 | 107.0 |
| IV(interaction) (all) | 0.085 | 6.911 | 3241.178 | 46801.7 | 5.6 | 84580.8 |
| SE <= 100 SE(ITT) (4826) | -0.003 | 0.639 | 2.884 | 351.4 | 5.8 | 624.0 |
| SE <= 10 SE(ITT) (4380) | -0.007 | 0.425 | 0.718 | 68.7 | 6.4 | 221.0 |
| p < 0.05 (987) | -0.016 | 0.353 | 0.373 | 5.6 | 10.7 | 121.6 |

**Scenario E; compliance scenario 4a**

| Analysis method | Mean of estimates | Empirical SE | Model-based SE | % bias in model-based SE | Power | Percentage increase in precision (ITT vs alternative) |
| --- | --- | --- | --- | --- | --- | --- |
| Intention to treat | 0.057 | 0.227 | 0.228 | 0.5 | 33.7 |  |
| Per protocol | 0.003 | 0.268 | 0.271 | 1.1 | 19.4 | 38.9 |
| IPW (all) | 0.004 | 0.289 | 0.296 | 2.6 | 16.7 | 61.4 |
| IPW (4987) | 0.004 | 0.288 | 0.296 | 2.9 | 16.7 | 60.5 |
| IV(Bayes) centred precise prior | -0.004 | 0.303 | 0.321 | 5.9 | 14.0 | 77.9 |
| IV(Bayes) centred vague prior | -0.004 | 0.303 | 0.398 | 31.3 | 8.2 | 77.9 |
| IV(Bayes) miscentred precise prior | -0.081 | 0.316 | 0.321 | 1.7 | 10.4 | 92.8 |
| IV(Bayes) miscentred vague prior | -0.081 | 0.316 | 0.398 | 25.8 | 7.0 | 93.4 |
| IV(interaction) (all) | -0.226 | 14.643 | 6464.705 | 44047.9 | 3.1 | 415270.4 |
| SE <= 100 SE(ITT) (4628) | 0.046 | 0.910 | 3.967 | 336.0 | 3.3 | 1503.7 |
| SE <= 10 SE(ITT) (3650) | 0.023 | 0.507 | 0.942 | 85.9 | 4.2 | 397.9 |
| p < 0.05 (268) | 0.051 | 0.390 | 0.407 | 4.5 | 14.6 | 194.5 |

**Scenario E; compliance scenario 4b**

| Analysis method | Mean of estimates | Empirical SE | Model-based SE | % bias in model-based SE | Power | Percentage increase in precision (ITT vs alternative) |
| --- | --- | --- | --- | --- | --- | --- |
| Intention to treat | -0.002 | 0.232 | 0.232 | -0.2 | 24.5 |  |
| Per protocol | 0.070 | 0.273 | 0.270 | -0.9 | 27.1 | 38.3 |
| IPW (all) | 0.070 | 0.290 | 0.296 | 2.0 | 23.1 | 56.0 |
| IPW (4995) | 0.070 | 0.289 | 0.295 | 2.2 | 23.1 | 55.4 |
| IV(Bayes) centred precise prior | -0.006 | 0.326 | 0.344 | 5.4 | 13.0 | 97.8 |
| IV(Bayes) centred vague prior | -0.005 | 0.326 | 0.391 | 19.9 | 9.5 | 97.9 |
| IV(Bayes) miscentred precise prior | -0.001 | 0.337 | 0.344 | 2.1 | 13.4 | 110.9 |
| IV(Bayes) miscentred vague prior | -0.001 | 0.337 | 0.391 | 16.1 | 10.1 | 110.8 |
| IV(interaction) (all) | -0.425 | 21.786 | 2350.346 | 10688.2 | 3.3 | 881943.9 |
| SE <= 100 SE(ITT) (4694) | -0.007 | 0.726 | 3.504 | 382.6 | 3.5 | 879.8 |
| SE <= 10 SE(ITT) (3919) | 0.001 | 0.457 | 0.867 | 89.8 | 4.2 | 288.0 |
| p < 0.05 (297) | 0.030 | 0.344 | 0.379 | 10.0 | 9.1 | 120.2 |

## Treatment effect heterogeneity scenarios

**Moderate treatment effect heterogeneity by X, moderate difference in compliance**

| Analysis method | Mean of estimates | Empirical SE | Model-based SE | % bias in model-based SE | Type I error | Percentage increase in precision (ITT vs alternative) |
| --- | --- | --- | --- | --- | --- | --- |
| Intention to treat | -0.173 | 0.103 | 0.102 | -0.4 | 23.9 |  |
| Per protocol | -0.298 | 0.120 | 0.121 | 0.4 | 2.7 | 37.0 |
| IPW (all) | -0.299 | 0.127 | 0.129 | 1.5 | 2.4 | 53.6 |
| IPW (5000) | -0.299 | 0.127 | 0.129 | 1.5 | 2.4 | 53.6 |
| IV(Bayes) centred precise prior | -0.311 | 0.134 | 0.151 | 12.4 | 1.1 | 70.5 |
| IV(Bayes) centred vague prior | -0.310 | 0.135 | 0.243 | 80.2 | 0.1 | 72.1 |
| IV(Bayes) miscentred precise prior | -0.390 | 0.137 | 0.151 | 10.2 | 0.3 | 77.3 |
| IV(Bayes) miscentred vague prior | -0.390 | 0.137 | 0.243 | 77.1 | 0.1 | 78.5 |
| IV(interaction) (all) | -0.114 | 3.198 | 1931.201 | 60294.3 | 2.4 | 96805.7 |
| SE <= 100 SE(ITT) (4952) | -0.112 | 0.345 | 0.766 | 122.3 | 2.5 | 1026.5 |
| SE <= 10 SE(ITT) (4749) | -0.143 | 0.243 | 0.303 | 24.8 | 2.6 | 459.7 |
| p < 0.05 (3403) | -0.172 | 0.212 | 0.226 | 6.4 | 3.5 | 326.6 |

**Moderate treatment effect heterogeneity by X, large difference in compliance**

| Analysis method | Mean of estimates | Empirical SE | Model-based SE | % bias in model-based SE | Type I error | Percentage increase in precision (ITT vs alternative) |
| --- | --- | --- | --- | --- | --- | --- |
| Intention to treat | -0.134 | 0.103 | 0.102 | -0.5 | 37.1 |  |
| Per protocol | -0.293 | 0.124 | 0.123 | -1.4 | 2.8 | 46.6 |
| IPW (all) | -0.299 | 0.130 | 0.132 | 1.4 | 2.1 | 59.9 |
| IPW (5000) | -0.299 | 0.130 | 0.132 | 1.4 | 2.1 | 59.9 |
| IV(Bayes) centred precise prior | -0.318 | 0.126 | 0.171 | 35.1 | 0.3 | 51.5 |
| IV(Bayes) centred vague prior | -0.318 | 0.128 | 0.379 | 196.5 | 0.0 | 55.1 |
| IV(Bayes) miscentred precise prior | -0.467 | 0.129 | 0.171 | 31.9 | 0.0 | 58.9 |
| IV(Bayes) miscentred vague prior | -0.467 | 0.131 | 0.379 | 189.9 | 0.0 | 62.2 |
| IV(interaction) (all) | -0.141 | 0.230 | 0.250 | 8.9 | 7.7 | 399.8 |
| SE <= 100 SE(ITT) (5000) | -0.141 | 0.230 | 0.250 | 8.9 | 7.7 | 399.8 |
| SE <= 10 SE(ITT) (4999) | -0.142 | 0.226 | 0.228 | 0.9 | 7.7 | 385.0 |
| p < 0.05 (4990) | -0.142 | 0.226 | 0.227 | 0.6 | 7.7 | 382.6 |

**Large treatment effect heterogeneity by X, moderate difference in compliance**

| Analysis method | Mean of estimates | Empirical SE | Model-based SE | % bias in model-based SE | Type I error | Percentage increase in precision (ITT vs alternative) |
| --- | --- | --- | --- | --- | --- | --- |
| Intention to treat | -0.179 | 0.103 | 0.103 | 0.2 | 21.0 |  |
| Per protocol | -0.296 | 0.121 | 0.121 | -0.0 | 3.0 | 40.3 |
| IPW (all) | -0.300 | 0.125 | 0.127 | 1.7 | 2.8 | 49.2 |
| IPW (5000) | -0.300 | 0.125 | 0.127 | 1.7 | 2.8 | 49.2 |
| IV(Bayes) centred precise prior | -0.319 | 0.134 | 0.151 | 12.5 | 1.3 | 71.6 |
| IV(Bayes) centred vague prior | -0.319 | 0.135 | 0.242 | 79.3 | 0.2 | 72.9 |
| IV(Bayes) miscentred precise prior | -0.398 | 0.137 | 0.151 | 10.0 | 0.4 | 79.7 |
| IV(Bayes) miscentred vague prior | -0.398 | 0.138 | 0.242 | 75.7 | 0.1 | 80.1 |
| IV(interaction) (all) | 0.070 | 1.442 | 52.927 | 3569.2 | 4.2 | 19681.9 |
| SE <= 100 SE(ITT) (4939) | 0.069 | 0.430 | 0.847 | 96.9 | 4.2 | 1661.2 |
| SE <= 10 SE(ITT) (4632) | 0.010 | 0.282 | 0.362 | 28.7 | 4.5 | 653.5 |
| p < 0.05 (3407) | -0.046 | 0.237 | 0.269 | 13.6 | 6.0 | 434.0 |

**Large treatment effect heterogeneity by X, large difference in compliance**

| Analysis method | Mean of estimates | Empirical SE | Model-based SE | % bias in model-based SE | Type I error | Percentage increase in precision (ITT vs alternative) |
| --- | --- | --- | --- | --- | --- | --- |
| Intention to treat | -0.151 | 0.101 | 0.103 | 1.6 | 30.3 |  |
| Per protocol | -0.286 | 0.122 | 0.123 | 1.3 | 3.3 | 45.0 |
| IPW (all) | -0.299 | 0.125 | 0.130 | 3.9 | 2.1 | 53.2 |
| IPW (5000) | -0.299 | 0.125 | 0.130 | 3.9 | 2.1 | 53.2 |
| IV(Bayes) centred precise prior | -0.339 | 0.124 | 0.171 | 37.8 | 0.1 | 51.4 |
| IV(Bayes) centred vague prior | -0.339 | 0.127 | 0.380 | 198.8 | 0.0 | 58.1 |
| IV(Bayes) miscentred precise prior | -0.488 | 0.127 | 0.171 | 34.6 | 0.0 | 58.7 |
| IV(Bayes) miscentred vague prior | -0.488 | 0.130 | 0.380 | 193.3 | 0.0 | 64.2 |
| IV(interaction) (all) | 0.020 | 0.255 | 0.273 | 7.1 | 19.0 | 534.4 |
| SE <= 100 SE(ITT) (5000) | 0.020 | 0.255 | 0.273 | 7.1 | 19.0 | 534.4 |
| SE <= 10 SE(ITT) (4993) | 0.018 | 0.247 | 0.252 | 2.2 | 19.0 | 494.5 |
| p < 0.05 (4990) | 0.018 | 0.247 | 0.252 | 2.1 | 19.0 | 494.0 |

**Moderate treatment effect heterogeneity by U, moderate difference in compliance**

| Analysis method | Mean of estimates | Empirical SE | Model-based SE | % bias in model-based SE | Type I error | Percentage increase in precision (ITT vs alternative) |
| --- | --- | --- | --- | --- | --- | --- |
| Intention to treat | -0.172 | 0.100 | 0.100 | -0.3 | 24.6 |  |
| Per protocol | -0.238 | 0.117 | 0.117 | -0.6 | 7.8 | 37.5 |
| IPW (all) | -0.237 | 0.126 | 0.129 | 2.2 | 6.6 | 58.4 |
| IPW (5000) | -0.237 | 0.126 | 0.129 | 2.2 | 6.6 | 58.4 |
| IV(Bayes) centred precise prior | -0.310 | 0.132 | 0.148 | 12.0 | 1.3 | 73.6 |
| IV(Bayes) centred vague prior | -0.310 | 0.133 | 0.241 | 81.9 | 0.3 | 75.5 |
| IV(Bayes) miscentred precise prior | -0.390 | 0.136 | 0.148 | 8.8 | 0.4 | 84.2 |
| IV(Bayes) miscentred vague prior | -0.390 | 0.136 | 0.241 | 77.0 | 0.1 | 84.9 |
| IV(interaction) (all) | 0.348 | 42.224 | 7543.783 | 17766.0 | 0.2 | 1.8e+07 |
| SE <= 100 SE(ITT) (4388) | -0.306 | 0.621 | 2.106 | 239.0 | 0.2 | 3750.8 |
| SE <= 10 SE(ITT) (3003) | -0.313 | 0.305 | 0.512 | 67.9 | 0.3 | 825.4 |
| p < 0.05 (245) | -0.323 | 0.224 | 0.229 | 2.4 | 2.0 | 398.6 |

**Moderate treatment effect heterogeneity by U, large difference in compliance**

| Analysis method | Mean of estimates | Empirical SE | Model-based SE | % bias in model-based SE | Type I error | Percentage increase in precision (ITT vs alternative) |
| --- | --- | --- | --- | --- | --- | --- |
| Intention to treat | -0.137 | 0.100 | 0.099 | -0.1 | 37.5 |  |
| Per protocol | -0.176 | 0.118 | 0.117 | -0.8 | 18.4 | 40.9 |
| IPW (all) | -0.175 | 0.126 | 0.130 | 2.9 | 14.9 | 60.8 |
| IPW (5000) | -0.175 | 0.126 | 0.130 | 2.9 | 14.9 | 60.8 |
| IV(Bayes) centred precise prior | -0.322 | 0.123 | 0.168 | 36.3 | 0.3 | 53.8 |
| IV(Bayes) centred vague prior | -0.321 | 0.126 | 0.379 | 201.0 | 0.0 | 59.7 |
| IV(Bayes) miscentred precise prior | -0.472 | 0.127 | 0.168 | 32.7 | 0.0 | 62.5 |
| IV(Bayes) miscentred vague prior | -0.471 | 0.129 | 0.379 | 194.7 | 0.0 | 66.8 |
| IV(interaction) (all) | -1.809 | 125.464 | 15397.686 | 12172.6 | 0.0 | 1.6e+08 |
| SE <= 100 SE(ITT) (4145) | -0.320 | 1.018 | 2.852 | 180.1 | 0.0 | 10361.8 |
| SE <= 10 SE(ITT) (1828) | -0.316 | 0.397 | 0.638 | 60.5 | 0.0 | 1492.7 |
| p < 0.05 (257) | -0.309 | 0.309 | 0.350 | 13.3 | 0.0 | 863.4 |

**Large treatment effect heterogeneity by U, moderate difference in compliance**

| Analysis method | Mean of estimates | Empirical SE | Model-based SE | % bias in model-based SE | Type I error | Percentage increase in precision (ITT vs alternative) |
| --- | --- | --- | --- | --- | --- | --- |
| Intention to treat | -0.179 | 0.098 | 0.099 | 0.3 | 22.6 |  |
| Per protocol | -0.246 | 0.115 | 0.115 | -0.0 | 6.9 | 36.0 |
| IPW (all) | -0.247 | 0.123 | 0.127 | 3.2 | 5.7 | 57.3 |
| IPW (5000) | -0.247 | 0.123 | 0.127 | 3.2 | 5.7 | 57.3 |
| IV(Bayes) centred precise prior | -0.319 | 0.129 | 0.146 | 13.2 | 1.1 | 72.8 |
| IV(Bayes) centred vague prior | -0.319 | 0.130 | 0.240 | 84.3 | 0.2 | 74.8 |
| IV(Bayes) miscentred precise prior | -0.398 | 0.133 | 0.146 | 10.1 | 0.2 | 82.7 |
| IV(Bayes) miscentred vague prior | -0.398 | 0.134 | 0.240 | 79.4 | 0.1 | 85.1 |
| IV(interaction) (all) | 0.161 | 33.805 | 5450.390 | 16022.9 | 0.1 | 1.2e+07 |
| SE <= 100 SE(ITT) (4380) | -0.324 | 0.613 | 2.131 | 247.8 | 0.1 | 3785.4 |
| SE <= 10 SE(ITT) (2889) | -0.322 | 0.287 | 0.489 | 70.4 | 0.1 | 753.7 |
| p < 0.05 (283) | -0.320 | 0.201 | 0.228 | 13.6 | 0.7 | 318.1 |

**Large treatment effect heterogeneity by U, large difference in compliance**

| Analysis method | Mean of estimates | Empirical SE | Model-based SE | % bias in model-based SE | Type I error | Percentage increase in precision (ITT vs alternative) |
| --- | --- | --- | --- | --- | --- | --- |
| Intention to treat | -0.149 | 0.097 | 0.098 | 1.0 | 34.0 |  |
| Per protocol | -0.190 | 0.115 | 0.115 | 0.3 | 15.8 | 39.3 |
| IPW (all) | -0.189 | 0.123 | 0.128 | 4.0 | 12.8 | 61.3 |
| IPW (5000) | -0.189 | 0.123 | 0.128 | 4.0 | 12.8 | 61.3 |
| IV(Bayes) centred precise prior | -0.336 | 0.120 | 0.167 | 39.1 | 0.1 | 53.0 |
| IV(Bayes) centred vague prior | -0.337 | 0.123 | 0.378 | 207.7 | 0.0 | 60.2 |
| IV(Bayes) miscentred precise prior | -0.486 | 0.124 | 0.167 | 35.1 | 0.0 | 61.9 |
| IV(Bayes) miscentred vague prior | -0.486 | 0.126 | 0.378 | 199.1 | 0.0 | 69.1 |
| IV(interaction) (all) | -0.230 | 81.283 | 17796.768 | 21794.7 | 0.0 | 7.0e+07 |
| SE <= 100 SE(ITT) (4127) | -0.354 | 0.952 | 2.644 | 177.9 | 0.0 | 9493.3 |
| SE <= 10 SE(ITT) (1914) | -0.338 | 0.397 | 0.629 | 58.5 | 0.1 | 1567.7 |
| p < 0.05 (262) | -0.314 | 0.317 | 0.357 | 12.7 | 0.4 | 965.2 |
